# The Intramolecular Povarov Tool in the Construction of Fused Nitrogen-Containing Heterocycles

**DOI:** 10.1007/s41061-023-00428-7

**Published:** 2023-05-30

**Authors:** Carme Masdeu, Jesús M. de los Santos, Francisco Palacios, Concepción Alonso

**Affiliations:** Departamento de Química Orgánica I, Facultad de Farmacia and Centro de Investigación Lascaray (Lascaray Research Center), Universidad del País Vasco/Euskal Herriko Unibertsitatea (UPV/EHU), Paseo de la Universidad 7, 01006 Vitoria-Gasteiz, Spain

**Keywords:** Povarov reaction, Intramolecular, Fused nitrogenated heterocycles, [4 + 2]-cycloaddition

## Abstract

Nitrogen heterocycles are part of the structure of natural products and agents with important biological activity, such as antiviral, antibiotic, and antitumor drugs. For this reason, heterocyclic compounds are one of today’s most desirable synthetic targets and the Povarov reaction is a powerful synthetic tool for the construction of highly functionalized heterocyclic systems. This process involves an aromatic amine, a carbonyl compound, and an olefin or acetylene to give rise to the formation of a nitrogen-containing heterocycle. This review illustrates advances in the synthetic aspects of the intramolecular Povarov reaction for the construction of intricate nitrogen-containing polyheterocyclic compounds. This original review presents research done in this field, with references to important works by internationally relevant research groups on this current topic, covering the literature from 1992 to 2022. The intramolecular Povarov reactions are described here according to the key processes involved, using different combinations of aromatic or heteroaromatic amines, and aliphatic, aromatic, or heteroaromatic aldehydes. Some catalytic reactions promoted by transition metals are detailed, as well as the oxidative Povarov reaction and some asymmetric intramolecular Povarov processes.

## Introduction

Nitrogen-containing heterocycles are present in many natural products and agents with important biological activity, such as antiviral, antibiotic, and antitumor drugs [1–5]. For this reason, the synthesis of *N*-heterocycles and their derivatives has always been an attractive topic in organic synthesis. Food and Drug Administration (FDA) databases reveal the structural importance of nitrogen-based heterocycles in drug design, considering that a large amount of small-molecule drugs contain a nitrogen heterocycle [6, 7]. In addition, *N*-heterocyclic skeletons are used as building blocks for a number of new drug candidates, due to the ability of the nitrogen atom to readily form hydrogen bonds with biological targets [8]. Hence, in the drug discovery process, the development of practical synthetic routes to access these structural motifs in the simplest possible way is an important goal for synthetic and medicinal chemists.

**Fig. 1:**
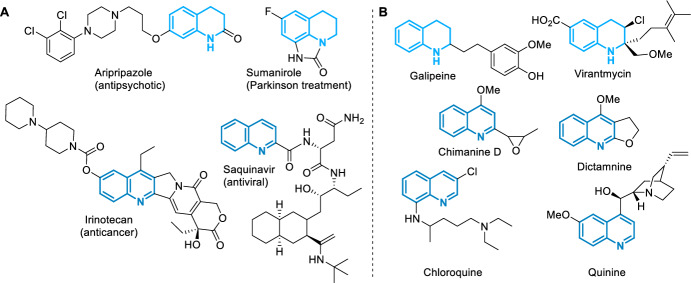
**A** Some marketed synthetic THQs and QUINs and their application. **B** Some bioactive THQs and QUINs from natural sources

One of the most straightforward and versatile processes for the preparation of heterocyclic nitrogen compounds is the Povarov reaction. Especially this reaction allows the preparation of tetrahydroquinolines (THQs) and their more aromatized analogs, the quinolines (QUINs), one of the most relevant nitrogen-containing heterocycles (Fig. 1) [9–12].

**Scheme 1:**
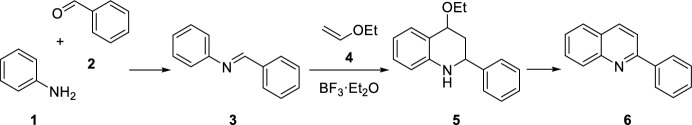
First example described by Povarov

This process was first reported in the 1960s, when Povarov et al. described the formation of two new C–C bonds from *N*-arylimines **3** with vinyl enolethers **4** under medium-strength Lewis acid catalysis (BF_3_·OEt_2_), thus obtaining substituted THQs **5** that were oxidized to the corresponding QUIN **6** (Scheme 1) [13].

Since Povarov’s first reaction, several reports indicated the versatility of this tool for the synthesis of nitrogen heterocycles [14, 15]. In general, in this process an aromatic amine **1**, a carbonyl compound **2**, and an olefin participate to give rise to the formation of a nitrogen-containing heterocycle. This hetero-Diels–Alder reaction represents a powerful tool for the construction of carbon–carbon and carbon–heteroatom bonds by generating three stereocenters in one step showing high regio- and diastereoselectivity. In cases where mixtures of diastereoisomers are observed, the ratio of *endo*/*exo* diastereoisomers formed is modulated and determined by the catalyst and solvent used in the process. Moreover, some developed catalytic enantioselective methods have been reviewed recently [16]. Two types of mechanism for this reaction have been described in the literature. Kobayashi et al. [17] suggested a stepwise mechanism via a cationic intermediate for the reaction catalyzed by rare earth triflates (Ln(OTf)_3_). On the other hand, Palacios et al. [18] in their combined computational and experimental study using BF_3_·OEt_2_ (the same Lewis acid as Povarov), as well as Xu et al. [19] in their work support the hypothesis that the cycloaddition occurs via an asynchronous concerted mechanism.

Apart from the mechanistic aspects, throughout the development and study of the Povarov reaction, other types of variables have been analyzed, such as: (a) the use of different catalysts in the reaction, both Lewis and Brønsted acids; (b) the nature of the dienophiles, olefins, acetylenes, etc.; (c) the design of the step-by-step or multicomponent protocols. These aspects have been extensively covered in excellent general reviews of this methodology in the recent literature [14–16, 20, 21].

**Scheme 2:**
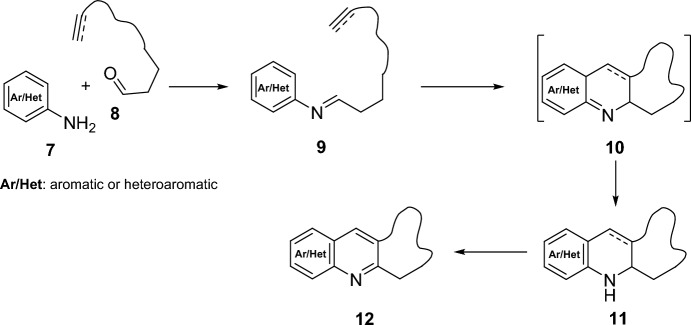
Intramolecular Povarov reaction

A particular case of a multicomponent reaction is the intramolecular Povarov reaction. For this purpose, aldimines present both, diene and dienophile functionality, in their structure. In general terms, intramolecular Povarov reaction can be explained as a formal intramolecular [4 + 2] cycloaddition reaction of aldimines **9**, obtained by condensation between aromatic or heteroaromatic amines **7** and aldehydes **8** functionalized with double or triple bonds (Scheme 2). The initial adduct **10** obtained by the intramolecular cycloaddition of aldimine **9**, followed by a double-bond tautomerization would generate the heteroaromatic compound **11** whose subsequent aromatization would give rise to derivatives **12**.

**Scheme 3:**
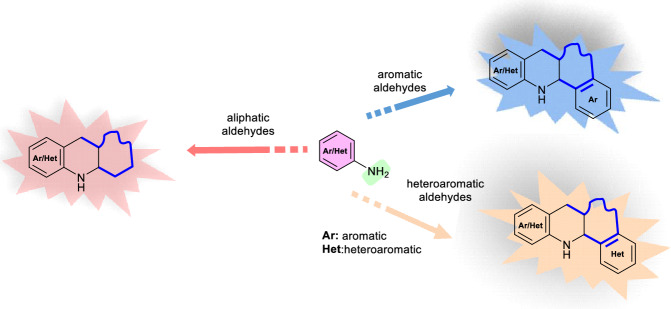
Scope of intramolecular Povarov reaction

Therefore, the intramolecular version of the Povarov reaction allows the generation of fused rings of high molecular complexity for the preparation of a wide variety of heterocyclic compounds. Skeletons of different sizes and with various condensed cycles can be obtained depending on the different combinations of aromatic or heteroaromatic amines with aliphatic, aromatic or heteroaromatic aldehydes (Scheme 3).

The aim of this review is to illustrate the advances in the synthetic aspects of the intramolecular Povarov reaction for the construction of intricate nitrogen-fused polyheterocyclic compounds. The revision is intended to be a comprehensive, authoritative, critical, and accessible review of general interest to the chemical community, as it contains a broad overview of published data on the intramolecular Povarov reaction covering the literature from 1992 to 2022, described according to the key processes involved, combining different aromatic/heteroaromatic amines and aliphatic/aromatic/heteroaromatic aldehydes. Some catalytic reactions promoted by transition metals are detailed, as well as the oxidative Povarov reaction and some asymmetric intramolecular Povarov processes. In addition, the effect of catalysts and solvents on the preparation of the final products will be examined, reflecting the synthetic potential of this strategy.

## Aromatic Amines and Aliphatic Aldehydes

### Aliphatic Alkene-Tethered Aldehydes

**Scheme 4:**
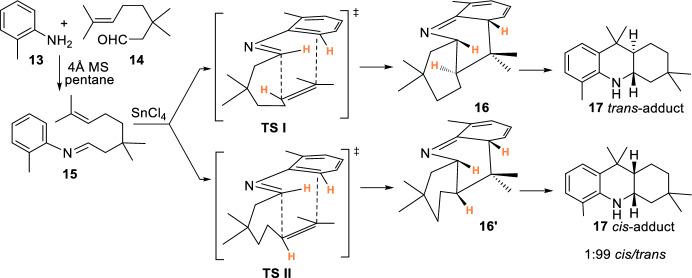
Mechanistic approaches to form *trans*- and *cis*-adducts **17**

Laschat’s group developed an intramolecular hetero-Diels–Alder reaction of *N*-aryl imines derived from aliphatic aldehydes tethered to non-activated olefins to afford 1,2,3,4,4a,9,9a,10-octahydroacridine (OHA) derivatives in high yields [22, 23]. The mechanism of this reaction could be explained either by concerted [4 + 2]-cycloaddition or by a multistep reaction via ionic intermediates. However, the authors suggested the concerted cycloaddition mechanism that would explain better the stereochemistry of the products. Then, treatment of imine **15**, obtained from 2-methylaniline **13** and 3,3-dimethylcitronellal **14**, with SnCl_4_ as Lewis acid afforded the *trans*-1,2,3,4,4a,9,9a,10-octahydroacridine **17** with a good yield (91%) and 1/99 *cis*/*trans*-diastereoselectivity. The formation of reaction compounds may be rationalized by cycloaddition through transition states **TSI** and **TSII**, which would lead to the formation of the *trans*- and *cis*-derivatives **17**, respectively (Scheme 4).

**Scheme 5:**
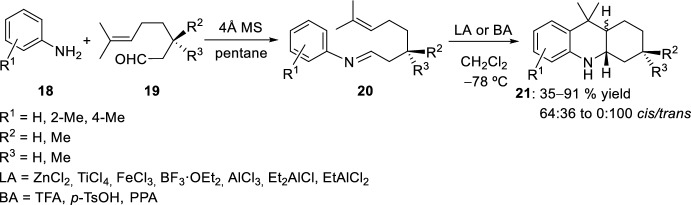
Synthesis of octahydroacridines **21** by using Lewis or Brønsted acids

Experimentally, both the reactivity and the *cis*/*trans-*selectivity of acridine derivatives **21** depended mainly on the substitution pattern at position 3 of the cyclization precursor **19**, where steric bulk at C-3 favored the formation of the *trans*-product (Scheme 5). The successive addition of the Lewis acid and the aldehydes **19** to a precooled solution (−78 °C) of the amines **18** was also investigated with yields and *cis*/*trans*-ratios quite similar to the cyclization when isolated imines **20** were used. A broad range of Lewis (LA) or Brønsted (BA) acids can catalyze the formation of octahydroacridines **21**, and the selectivity was found to be more dependent on the substrate structure than on the type of catalyst used.

**Scheme 6:**
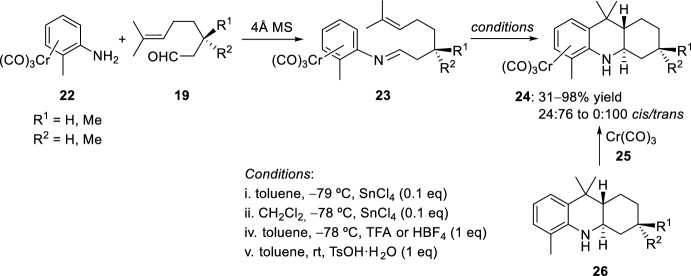
Synthesis of (octahydroacridine)chromium tricarbonyl complexes **25**

This process has been extended to chromium complexes. When starting from chromiun tricarbonylamine **22**, the highly *trans*-selective preparation of (octahydroacridine)chromium complexes **24** was accomplished through the intramolecular [4 + 2]-cycloaddition of the (imino-arene)chromium complexes **23** (Scheme 6) [24]. Different conditions were used. For example, the reaction of the imino complexes **23** with catalytic amounts of SnCl_4_ (10 mol%) gave (1,2,3,4,4a,9,9a,l0-octahydroacridine)chromium tricarbonyl complexes **24** in high yields and stereoselectivity toward the *trans*-products, which seems to agree with a concerted hetero-Diels–Alder type mechanism. These (octahydrocridine)chromium complexes **24** could be also prepared by direct complexation of octahydroacridines **26** with chromium tricarbonyl **25** [25].

**Scheme 7:**
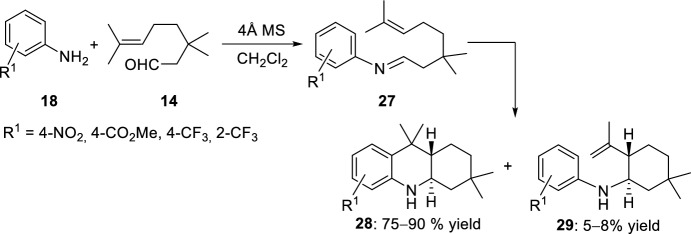
Synthesis of octahydroacridines **28** and/or amines **29** with molecular sieves as activating agents

A major drawback in cyclization and cycloaddition reactions of imines is the necessary activation; therefore, Laschat et al. [26] studied the reaction of anilines with *para*- and *ortho*-electron-withdrawing substituents with 3-methylcitronellal **14** in the presence of molecular sieves. The authors observed that the reaction proceeded differently depending largely on the type of molecular sieves used (Scheme 7). When powdered molecular sieves were used, anilines **18** gave very pure imines **27** in almost quantitative yield after 15 min. However, while the 4-nitroaniline **18** (R^1^ = 4-NO_2_) could not be converted to the imine **27** with powdered molecular sieves, when molecular sieve beads were used the formation of a mixture of the *trans*-cyclization products **28** and **29** was observed with other anilines.

**Scheme 8:**
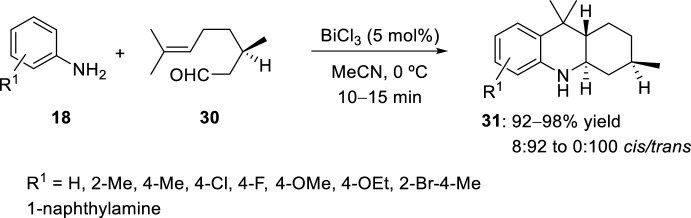
Synthesis of octahydroacridines **31** using bismuth(III) chloride as catalyst

In previous works, a variety of Lewis or Brønsted strong acids has been used for the preparation of octahydroacridine derivatives. Sabitha et al. [27] studied the intramolecular hetero-Diels–Alder reaction of (*R*)-citronellal **30** and anilines **18** when bismuth (III) chloride was used (Scheme 8). As authors stated, the temperature of the reaction medium seems to play a key role in determining the *cis*/*trans* ratio formation, and in this case, the reaction with a catalytic amount of BiCl_3_ at 0 °C in acetonitrile proceeds in a highly stereoselective fashion giving *trans*-products **31** diastereoselectively. These *trans*-adducts **31** were obtained exclusively when non-substituted amines **18** (R^1^ = H) were used.

**Scheme 9:**
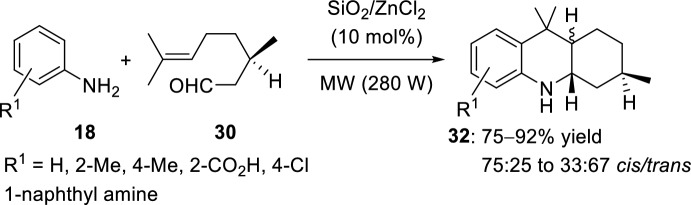
Synthesis of octahydroacridines **32** using a solid-supported catalyst

A solid-supported catalyst (SiO_2_/ZnCl_2_), under MW irradiation and without any solvent, has also been used for the synthesis of octahydroacridines (Scheme 9) [28]. Thus, an environmentally friendly and efficient method consisting on a facile imino-Diels–Alder reaction from *N*-aryl amines **18** and (*R*)-citronellal **30** has been developed and corresponding octahydroacridine derivatives **32** have been obtained in good yields.

**Scheme 10:**
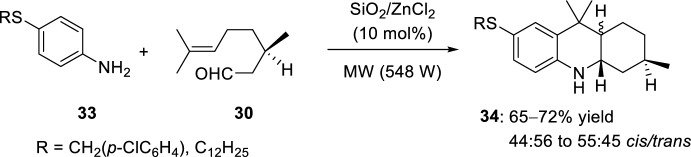
Synthesis of octahydroacridine derivatives **34** using a solid-supported catalyst and thio-functionalized anilines

Six years later, the same group reported the synthesis of several acridine derivatives with sulfur substituents by using the same catalyst, (*R*)-citronellal **30** and thio-functionalized anilines **33**. In this case, octahydroacridines **34** were obtained in moderate yields and poor diastereoselectivity, obtaining almost stoichiometric mixtures of *cis*- and *trans*-diastereoisomers (Scheme 10) [29].

**Scheme 11:**
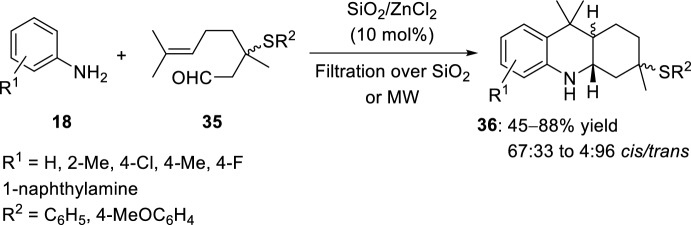
Synthesis of octahydroacridine derivatives **36** with a solid-supported catalyst and thio-functionalized aldehydes

The intramolecular Povarov reaction mediated by solid-supported catalyst (SiO_2_/ZnCl_2_) at room temperature was achieved using 3-(arylthio)citronellal **35** and anilines **18** for the preparation of octahydroacridines **36** (Scheme 11) [29]. The best results were obtained when a mixture of aromatic amines **18** and 3-(phenylthio)citronellal **35** was stirred in the presence of SiO_2_/ZnCl_2_ (10 mol%) at room temperature, yielding the corresponding acridine derivatives **36** (Scheme 11). Regarding the stereochemistry of the ring fusion, the formation of a mixture of *trans*- and *cis*-adducts was observed, with good selectivity for the *trans*-fused 3-(phenylthio)octahydroacridines **36** in most of the cases. However, *o*-toluidine and 1-naphthylamine reacted with aldehyde **35** to afford mainly adducts with *cis*-selectivity (53:47–63:37 *cis*/*trans*).

**Scheme 12:**
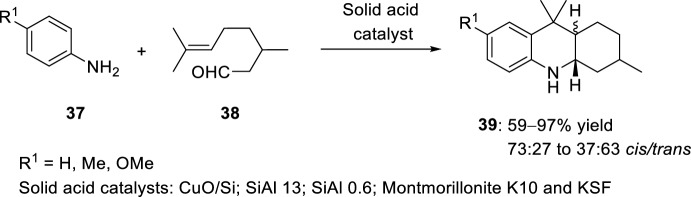
Synthesis of octahydroacridines **39** catalyzed by different solid acid catalysts

The synthesis of octahydroacridines **39** has been performed through intramolecular imino-Diels–Alder reaction starting from aniline **37** and citronellal **38** catalyzed by solid acid catalyst (Scheme 12) [30]. Several catalytic materials were studied, firstly, in the presence of pre-reduced Cu catalyst with molecular hydrogen, giving the corresponding octahydroacridines **39** as the only product in high yield. The use of the unreduced CuO/SiO_2_ (CuO/Si) catalyst at room temperature in the presence of air gave comparable results. Other solid acids can be used in the synthesis of **39**, such as silica-alumina cracking catalysts with a 13% content of Al_2_O_3_ (SiAl 13) and a 0.6% alumina on silica (SiAl 0.6). Furthermore, two commercial acid-treated clays were also tested, namely Montmorillonite K10 and KSF. However moving from aniline **37** (R^1^ = H) to electron-rich amines **37** (R^1^ ≠ H), the selectivity of heterocycles **39** increased. In addition, differences in stereochemistry are evident. Pure Lewis solids (both CuO/silica and SiAl 0.6) promote 40/60 mixtures with a slight excess of *trans*-isomers. Conversely, the use of Brønsted acids, as Montmorillonite KSF, favor selectively the formation of the *cis*-isomer.

**Scheme 13:**
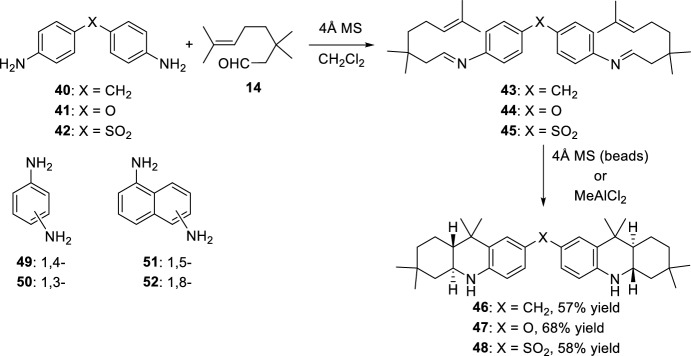
Synthesis of bis-octahydroacridines **46**–**48**

Based on the activation with molecular sieves, Laschat et al. studied the bis-cyclization of *N*-aryl diimines for the preparation of polycyclic ring systems [26]. In diamines with “separated” aromatic systems the reaction proceeded with molecular sieve beads. In this sense, when aryldiamines **40**–**42** were treated with 3-methylcitronellal **14** in the presence of 4 Å molecular sieve beads (Scheme 13), the biscyclization was complete after 24 h as determined by nuclear magnetic resonance (NMR) spectroscopy and corresponding compounds **46**–**48** were isolated. However, treatment of compound **40** (X = CH_2_) with aldehyde **14** in the presence of powdered molecular sieves gave, as expected, complete formation of the diimine **43** (X = CH_2_) after 15 min with no further cyclization. On the other hand, treatment of the isolated imine **43** with MeAlCl_2_ yielded the biscyclization product **46** (X = CH_2_). When the aromatic diamine has both functionalities in the same aromatic system (diamines **49**–**52**, Scheme 13), the biscyclization reaction was not possible neither with powered molecular sieves nor with 4 Å MS beads. This means that the presence of a second imino function in the same aromatic ring decreases the reactivity of the first one, so that a stronger activation, e.g., by Lewis acids, is required to obtain the biscyclization products. Moreover, only when diamines **49** and **52** are used are the corresponding biscycloadducts obtained, probably because the two amino functions should be separated from each other, avoiding unfavorable steric interactions. Therefore, after formation of corresponding bisimines from amines **49** and **52**, the treatment with 2.5 equivalents of MeAlC1_2_ gave the corresponding biscyclization products.

**Scheme 14:**
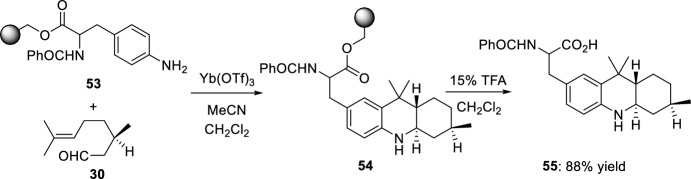
Solid-phase synthesis of octahydroacridine **55**

Exploiting the advantages of the solid-phase synthesis methodology, the preparation of octahydroacridines by concomitant formation of the imine and subsequent intramolecular capture of the alkene has been reported [31]. For example, when aniline resin **53** was reacted with (*R*)-citronellal **30** in the presence of Yb(OTf)_3_, the only product isolated after cleavage of the resin with TFA was octahydroacridine **55** in excellent yield as a single diastereoisomer (Scheme 14).

**Scheme 15:**
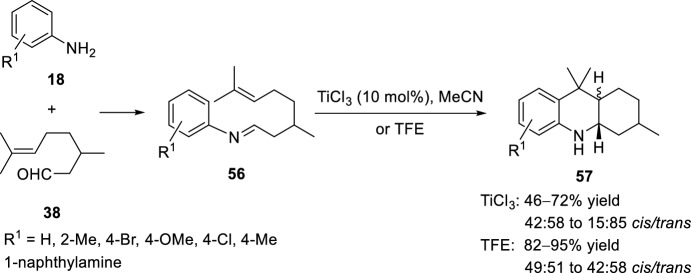
Synthesis of octahydroacridines **57** by fluorous phase synthesis

Fluorous phase synthesis was also applied for the preparation of octahydroacridines [32]. Intramolecular aza-Diels–Alder reaction of functionalized *N*-aryl imines **56**, produced in situ from aryl amines **18** and citronellal **38**, was carried out at room temperature in the presence of trifluoroethanol (TFE), without any additional catalyst. In parallel, same reactions were studied in the presence of 10 mol% TiCl_3_ (Scheme 15). Regarding the yields of the reactions, a greater amount of product **57** was obtained with the use of the fluorinated solvent; however, worse results were obtained in terms of the stereochemistry of the reaction, while TiCl_3_ afforded a diastereomeric excess of *trans*-derivatives **57** the use of TFE gave to equimolecular mixtures of *cis*- and *trans*-derivatives.

**Scheme 16:**
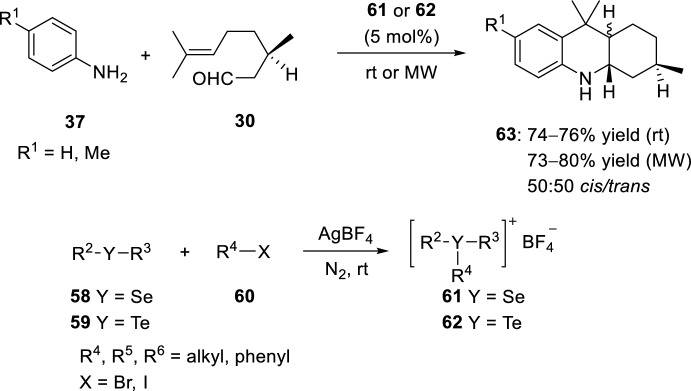
Synthesis of octahydroacridines **63** by using selenium- and tellurium-based ionic liquids

The use of Lewis acids for the preparation of octahydroacridines presents some drawbacks, such as long reaction times, the use of low temperatures (−78 °C), and organic solvents. As previously reported, molecular sieves and BiCl_3_ have been introduced, among other variations, as alternatives for cyclization promoters solving some of these limitations. Moreover, with the use of ionic liquids (IL), easily recoverable after the reaction, no polluting solvents or complementary catalysts are needed, as the ionic liquids perform this function. In this regard, selenium- and tellurium-based ionic liquids have been used as solvents and/or catalysts in the synthesis of octahydroacridines via the hetero-Diels–Alder reaction involving (*R*)-citronellal **30** and aryl amines **37** (Scheme 16) [33]. When using 5 mol% of ionic liquid **61** or **62** the expected products **63** are obtained in good yield. Moreover, to reduce the reaction times, microwave irradiation was used, the consumption of the starting products was observed in 6 min and the expected products **63** were also obtained in good yields. A *cis*/*trans*-mixture of diastereoisomers was obtained as determined by NMR spectroscopy.

**Scheme 17:**
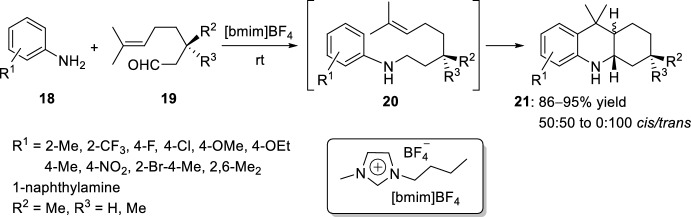
Synthesis of octahydroacridines **21** with ionic liquids

In the same way, other ionic liquids, such as 1-butyl-3-methylimidazolium tetrafluoroborate [bmim]BF_4_, 1-hexyl-3-methylimidazolium tetrafluoroborate [hmim]BF_4_, and 1-octyl-3-methylimidazolium tetrafluoroborate [octmim]BF_4_, have resulted suitable solvents to obtain octahydroacridine derivatives **21** (Scheme 17) [34]. The cycloaddition of aryl imines **20**, formed in situ from a wide range of anilines **18** and (*R*)-citronellal **19** (R^2^ = Me, R^3^ = H) or 3-methyl citronellal **19** (R^2^ = R^3^ = Me), exhibited improved reactivity in the ionic liquid, thus reducing reaction time and significantly improving the yield. For example, the treatment of aniline **18** (R^1^ = H) with (*R*)-citronellal **19** (R^2^ = Me, R^3^ = H) at room temperature, without the need for any additional catalyst, resulted in the formation of 3,9,9-trimethyl-1,2,3,4,4a,9,9a,10-octahydroacridine **21** in 95% yield as a mixture of *cis*- and *trans*-isomers.

**Scheme 18:**
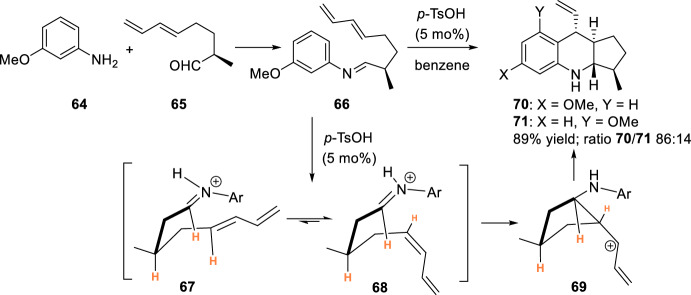
Synthesis of cyclopenta[*b*]quinolines **70** and **71**

Not only citronellal was used as a suitable aldehyde for intramolecular Povarov reaction with anilines. The synthesis of cyclopenta[*b*]quinoline **70** and **71**, as a part of isoschizozygane alkaloids, was accomplished by an intramolecular formal hetero-Diels–Alder Brønsted acid-catalyzed reaction from imine **66** obtained from unsaturated aldehyde **65** and aniline **64** (Scheme 18) [35]. In this case, the acid-catalyzed condensation of aromatic amine **64** with conjugated unsaturated aldehyde **65**, followed by the cycloaddition reaction is described as a good route for the preparation of cyclopenta[*b*]quinoline derivative **70** and **71**. The optimized conditions involved catalysis with 5 mol% of TsOH and provided 89% yield of an 86:14 mixture of **70** and **71** that could be readily separated by crystallization or column chromatography. The reaction is highly diastereoselective and produces adducts **70** and **71** with four contiguous stereocenters. The authors suggest in this case that the diastereoselectivity in adduct formation is determined by the nucleophilic attack of the diene on the iminium ion and directed by the C-3 stereocenter. Semiempirical calculations suggest that the closure of the cyclopentane ring would occur via a more stable intermediate **68** and give rise to **69** with a *trans* arrangement of the allylic cation and an amine. An alternative intermediate **67** is destabilized by the torsional interaction of the dienyl and imino moieties. The electrophilic aromatic substitution reaction between the allylic cation and the aniline in **69** will give rise to the more stable 1,2,4-trisubstituted arenes of structure **70** and **71**.

**Scheme 19:**
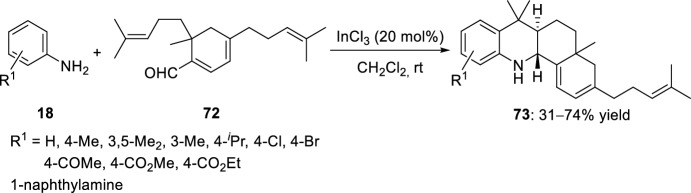
Preparation of octahydrobenzo[*c*]acridines **73**

Fused acridines have been prepared also by intramolecular Povarov reaction. 1,2,4-Trisubstituted cyclohexadienal **72**, obtained by self-condensation of citral in the presence of NaH, is a suitable carbonyl substrate in the Povarov reaction providing molecular complexity and structural diversity. In this way, octahydrobenzo[*c*]acridines **73** have been prepared by intramolecular Povarov reaction of substituted anilines **18** and aldehyde **72** catalyzed by InCl_3_ (Scheme 19) [36]. Methylene chloride was selected as the best solvent for this transformation, since ethyl ether, THF, or hexanes were found to be less efficient. The scope of substituted anilines **18** with a varied array of functional groups was studied, showing a strong reaction efficiency effect by steric and electronic factors. For instance, electron-withdrawing groups and sterically crowded anilines lead to acridine derivatives **73** in very low yield.

**Scheme 20:**
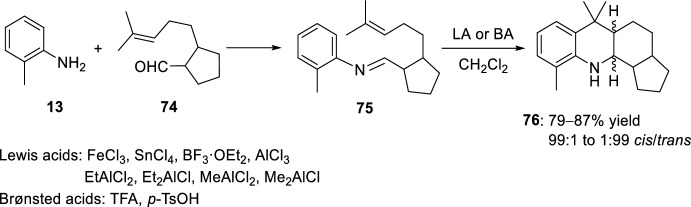
Synthesis of cyclopenta[*c*]acridine derivatives **76**

Condensation of aldehyde **74** with *o*-toluidine **13** afforded compound **75** whose subsequent intramolecular hetero-Diels–Alder reaction catalyzed by a Lewis or Brønsted acid produced diastereoselectively the cyclopenta[*c*]acridine derivatives **76** (Scheme 20) [37]. The formation of the *cis*- or *trans*-isomers was modulated depending on the Lewis or Brønsted acid used.

See Table 1 for the most representative examples of Sect. 2.1.

**Table 1:** Some examples of the intramolecular Povarov reaction between aromatic amines and aliphatic alkene-tethered aldehydes


Entry	R^1^	R^2^	R^3^	Catalyst	dr *cis/trans*	Yield (%)	References
1	H, 2-Me, 4-Me	H, Me		ZnCl_2_, TiCl_4_, FeCl_3_, BF_3_·OEt_2_, AlCl_3_, Et_2_AlCl, EtAlCl_2_, TFA, *p*-TsOH, PPA	64:36 to 0:100	35–91	[22, 23]
2	2-Me [Cr (CO)_3_]	H, Me		SnCl_4_, TFA, HBF_4_, TsOH·H_2_O	24:76 to 0:100	31–98	[24]
3	4-NO_2_, 4-CO_2_Me, 4-CF_3_, 2-CF_3_	Me		4 Å MS beads	0:100	75–90	[26]
4	H, 2-Me, 4-Me, 4-Cl, 4-F, 4-OMe, 4-OEt, 2-Br-4-Me, 1-naphthylamine	Me	H	BiCl_3_	8:92 to 0:100	92–98	[27]
5	H, 2-Me, 4-Me, 4-Cl, 2-CO_2_H, 1-naphthylamine	Me	H	Solid-supported SiO_2_/ZnCl_2_	75:25 to 33:67	75–92	[28]
6	4-SCH_2_(*p*-ClC_6_H_4_), 4-SC_12_H_25_	Me	H	Solid-supported SiO_2_/ZnCl_2_	44:56 to 55:45	65–72	[29]
7	Solid-supported substituent in *p*-position	Me	H	Yb(OTf)_3_	0:100	88	[31]
8	H, 4-Me	Me	H	Selenium- and tellurium-based ionic liquids	50:50	73–80	[33]
9	2-Me, 2-CF_3_, 4-F, 4-Cl, 4-OMe, 4-OEt, 4-Me, 4-NO_2_, 2-Br-4-Me, 2,6-Me_2_, 1-naphthylamine	Me	H, Me	[bmim]BF_4_	50:50 to 0:100	86–95	[34]

### Steroid- and Carbohydrate-Derived Aldehydes

**Scheme 21:**
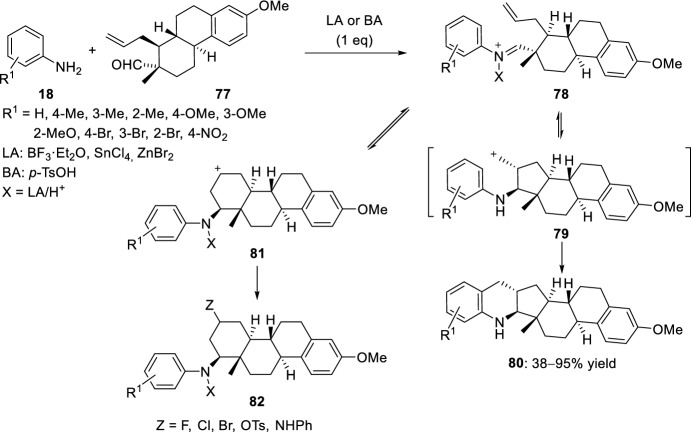
Synthesis of quinolines **80** condensed to a steroid skeleton

The intramolecular Povarov reaction with aliphatic aldehydes has been applied to the preparation of hybrid derivatives of tetrahydroquinolines condensed to a steroid skeleton by using Lewis or Brønsted acids. For this purpose, the reaction of the estrone derivative **77**, with an allyl and a formyl group in suitable positions, with different anilines **18** was studied (Scheme 21) [38, 39]. After the reaction of aldehyde **77** and anilines **18** and subsequent treatment with BF_3_·OEt_2_, two different cyclic products **80** and **82** were obtained. Although compound **80** is the formal Diels–Alder adduct, the authors indicated that this compound may be obtained in a two-step mechanism from the initially formed iminium ion **78**, which led to the carbocation **79** and then an electrophilic aromatic substitution to give **80**. However, the iminium ion in **78** might also react with the alkene moiety to afford the cation **81** which could be further transformed by the addition of a nucleophile into compound **82**.

**Scheme 22:**
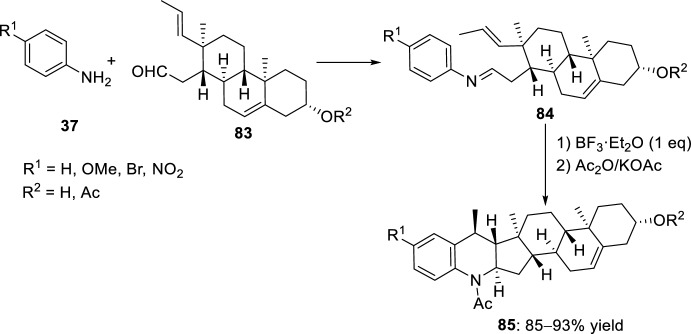
Synthesis of heterocycles **85** from steroid-derived aldehydes

In a similar manner, other aryl imino steroids **84** were prepared from the aldehyde steroid fragment **83** and various anilines **37** (Scheme 22) [40]. Afterwards, their intramolecular cyclization was studied via a Lewis acid-catalyzed reaction in the presence of BF_3_·OEt_2_. The nature of the substituent on the aniline influenced the reactivity since different tetrahydroquinoline derivatives **85** were formed from unsubstituted (R^1^ = H) or substituted (R^1^ = Br, OMe) anilines **37** followed by treatment with acetic acid anhydride/potassium acetate. However, when 4-nitroaniline (R^1^ = NO_2_) was used, a fluoro-d-homosteroid derivative was isolated, apparently through an intramolecular Prins reaction.

**Scheme 23:**
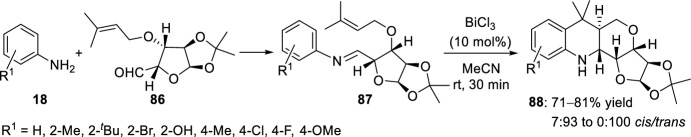
Synthesis of pyrano[4,3-*b*]quinolines **88** from *O*-allyl carbohydrate-derived aldehydes

Not only steroid-derived aldehydes have been used in intramolecular Povarov type cycloaddition reactions, as previously indicated, but also carbohydrate-derived aldehydes. Because natural carbohydrates, readily available and affordable, present a certain absolute stereochemistry, they are interesting substrates as chiral auxiliaries or chiral building blocks. Intramolecular hetero-Diels–Alder reactions of carbohydrate-derived aldehydes have been performed by Sabitha et al. [41]. In this work, pyrano[4,3-*b*]quinolines **88** have been prepared in a highly efficient and stereoselective way (Scheme 23). Aldimines **87** generated in situ from aromatic amines **18** and the *O*-allyl derivative of the d-glucose aldehyde **86** were treated in acetonitrile in the presence of a catalytic amount of BiCl_3_. This Lewis acid is the most suitable, since it can be used in substoichiometric amount (10 mol%), while other Lewis acids such as ZnCl_2_, FeCl_3_, ZrCl_4_, AlCl_3_, and BF_3_·OEt_2_ are needed in at least stoichiometric amounts. Cycloadducts **88** were obtained with high selectivity and good to excellent yields. In general, the reactions led to the formation of *trans*-isomers as major products, although small amounts of *cis*-isomers were observed. However, when a bulky group, such as *tert*-butyl (R^1^ = ^*t*^Bu), was present in the *ortho* position of the amine **18**, only the *trans*-adduct **88** was exclusively obtained.

**Scheme 24:**
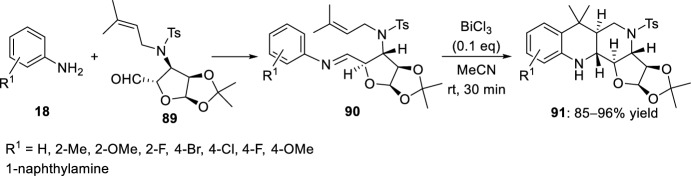
Synthesis of furo[3,2-*h*][1,6]naphthyridines **91** from *N*-allyl carbohydrate-derived aldehydes

On the basis of this protocol, the same group has performed the preparation of tetra- or pentacyclic furo[3,2-*h*][1,6]naphthyridine derivatives from anilines or 1-naphthylamine and a simple sugar derivative [42]. In this case, an *N*-prenylated sugar aldehyde **89** and different aromatic amines **18** were used in the condensation reaction to give the imines **90** (Scheme 24). Afterwards, intramolecular hetero-Diels–Alder reaction in the presence of bismuth(III) chloride as catalyst, under very mild conditions, was completed within 30 min to give the corresponding *trans*-fused products **91** stereoselectively and in good to excellent yields.

### Nitrogen- or Oxygen-Containing Aliphatic Alkene or Alkyne-Tethered Aldehydes

**Scheme 25:**
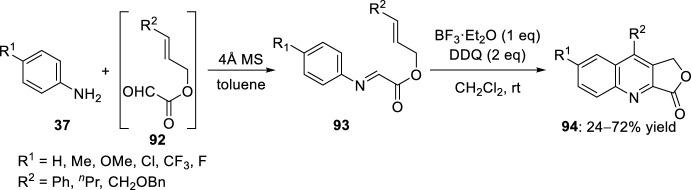
Quinoline-fused lactones **94** obtained by intramolecular Povarov reaction

Some functionalized aldehydes with side chains possessing alkene or alkyne linked to a heteroatom such as oxygen or nitrogen have been described, allowing the preparation of fused heterocycles. For example, aldimines **93** derived from condensation of aromatic amines **37** with glyoxylic acid-derived *O*-allyl ester **92** in toluene and in the presence of molecular sieves were subjected to Lewis acid-catalyzed intramolecular Povarov reaction (Scheme 25) [43]. Using 1 equivalent of BF_3_·OEt_2_ in CH_2_Cl_2_ at room temperature, a mixture of quinoline-fused lactones **94** and amines derived from the reduction of aldimines **93** was observed, instead of expected tetrahydroquinoline-fused lactone (Scheme 25). This result suggests that the imine **93** reacts as an oxidant to convert the expected tetrahydroquinoline into quinoline **94**, confirming that this oxidation proceeds faster or at the same rate as the intramolecular Povarov cycloaddition. However, the presence of an oxidant such as 2,3-dichloro-5,6-dicyano-*p*-benzoquinone (DDQ) afforded quinoline **94** in low to moderate yields. Two equivalents of DDQ were necessary to convert aldimines **93** into quinoline-fused-lactone **94** with better yields without any traces of amines derived from the reduction of aldimines **93**.

**Scheme 26:**
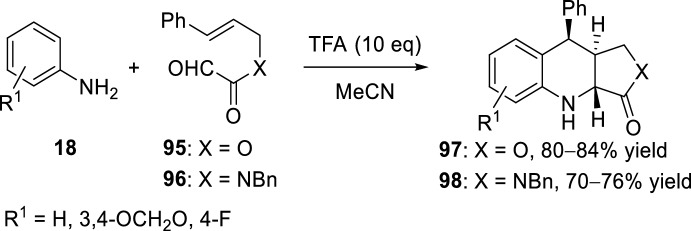
Intramolecular Povarov reaction with glyoxylic **95** or glyoxamide-derived aldehydes **96**

In a similar way, the glyoxylic acid-derived aldehydes **95** (X = O) react with anilines **18** in the presence of TFA to afford the tetrahydroquinoline lactones **97**. A stepwise process in the [4 + 2]-cyclization would provide the *trans*-diastereoisomer (Scheme 26) [44]. When the reaction was performed with the *N*-benzylglyoxamides **96** (X = NBn) the corresponding derivatives **98** were obtained. In these cyclizations, the *trans*-configuration of the major isomer was obtained (Scheme 26).

**Scheme 27:**
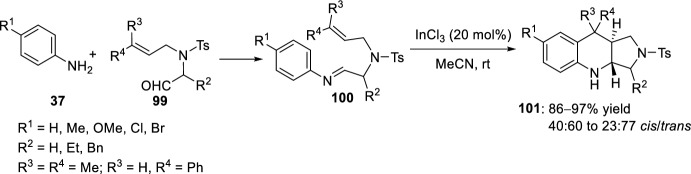
InCl_3_-catalyzed intramolecular Povarov reaction for the preparation of pyrroloquinolines **101**

This process has also been extended to α-amino acid-derived aldehydes. Raghunathan et al. [45, 46] described an efficient synthesis of unreported fused pyrroloquinolines through reaction of aldimines **100**, resulting from aromatic amines **37** and *N*-prenylated aliphatic aldehydes **99**, in a Lewis acid catalyzed intramolecular Povarov reaction. Aldehydes **99** and anilines **37** were subjected to the intramolecular Povarov reaction using a 20 mol% of InCl_3_ in MeCN (Scheme 27). Thus, after generation of the corresponding imine, this is trapped by an *N*-tethered prenyl moiety cyclizing intramolecularly to give the Povarov adducts **101** in excellent chemical yields and *trans*-selectivity (40:60–23:77 *cis*/*trans*). Pyrroloquinolines **101** exhibited good antibacterial activity toward six different bacterial strains with MIC values of 5 mM. Gyrase assays showed the potential of compounds **101** to bind to gyrase, preventing their gene expression [45, 46]. The same group reported the diastereoselective synthesis of *trans*-fused pyrroloquinolines **101** by the InCl_3_-promoted intramolecular Povarov reaction of aldimines resulting from the condensation of aromatic amines **37** and alkene-tethered aldehydes derived from (*S*)-phenylalanine **99** (R^2^ = Bn) [47]. The cycloaddition reaction resulted to be stereoselective, and *trans*-pyrroloquinolines **101** were obtained in 86–97% yield.

**Scheme 28:**
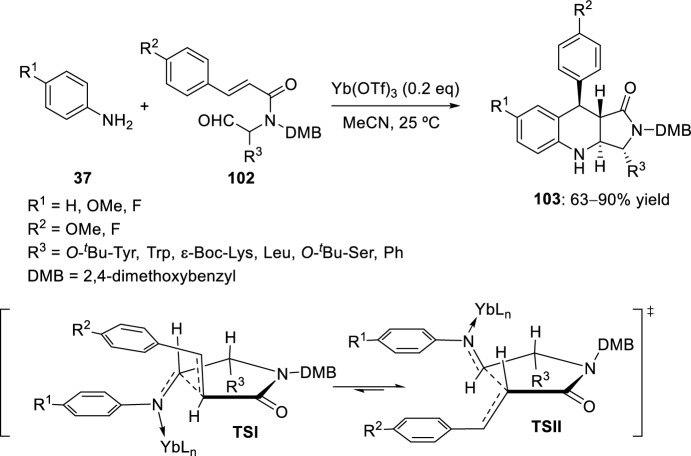
Ytterbium triflate-catalyzed Povarov reaction with *N*-cinnamoyl-α-aminoaldehydes

Cyclization of the imine formed from cinnamoylaminoaldehyde **102** derived from different amino acids with aniline derivatives **37** using the mild Lewis acid ytterbium triflate yielded the thermodynamically more stable *trans*-products **103** (Scheme 28). The stereoselectivity was explained by a stepwise mechanism involving the transition states **TSI** and **TSII** (Scheme 28). In the electrophilic attack of the ytterbium–imine complex, fewer steric interactions would occur between the R^3^ substituent and the aromatic ring of the aniline in **TSII**, maintaining the equatorial orientation of the aryl substituent in the subsequent closure of the second ring. The strategy of the cyclization from aminoaldehydes **102** was also transferred from solution to solid phase [44].

**Scheme 29:**
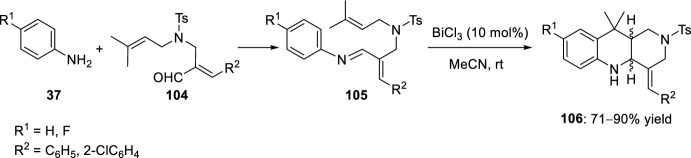
BiCl_3_-promoted intramolecular Povarov reaction with *N*-(prenylaminomethyl)cinnamaldehyde

Likewise, aldimines **105** prepared from *N*-(prenylaminomethyl)cinnamaldehydes **104** derived from Morita–Baylis–Hillman adducts of acrylates, and aromatic amines **37**, were subjected to an intramolecular cycloaddition reaction to furnish benzonaphthyridine derivatives **106** (Scheme 29) [48]. Several Lewis acids, for instance, Yb(OTf)_3_, InCl_3_, Sc(OTf)_3_, or BiCl_3_, or even Brønsted acids such as TFA, were used for this transformation; however, only in the presence of BiCl_3_ did the reaction proceed efficiently to afford azaheterocycles **106** in good to excellent yields, but as a diastereomeric mixture of *cis*- and *trans*-adducts.

**Scheme 30:**
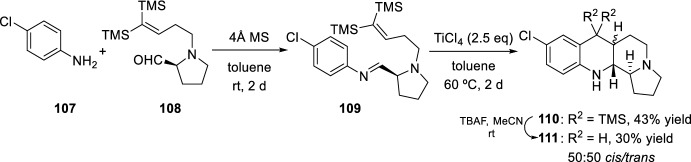
Synthesis of fused benzo[*b*]pyrrolo[1,2-*h*][1,7]naphthyridines **111**

Fused benzo[*b*]pyrrolo[1,2-*h*][1,7]naphthyridine heterocycles **111**, without a *gem*-dimethyl group, were prepared by taking vinyldisilane-terminated *N*-aryl imine **109** as a precursor obtained from aldehydes derived from l-prolinol **108** (Scheme 30) [49]. This process yielded new benzo[*b*]pyrrolo[1,2-*h*][1,7]naphthyridines **111** as a 50:50 mixture of diastereoisomers by Lewis acid-catalyzed cyclization of *N*-aryl imines **109** as a key step.

**Scheme 31:**
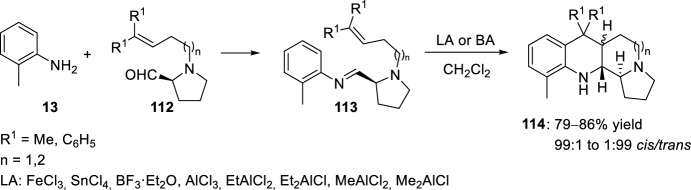
Intramolecular Povarov reaction of l-proline-derived aldehydes

Condensation of the l-proline-derived aldehydes **112** with *o*-toluidine **13** afforded compounds **113** whose subsequent intramolecular hetero-Diels–Alder reaction catalyzed by a Lewis or Brønsted acid produced diastereoselectively the benzo[*b*]pyrrolo[1,2-*h*][1,7]naphthyridine derivatives **114** (Scheme 31) [37, 50, 51]. This cycloaddition reaction, where a second nitrogen atom has been introduced, displayed a remarkable Lewis acid-dependent reversal of the diastereoselectivity. The formation of the *cis*- or *trans*-isomers was modulated depending on the Lewis or Brønsted acid used. When using FeCl_3_, SnCl_4_, BF_3_·OEt_2_, *p*-TsOH, TFA, AlCl_3_, and Et_2_AlCl *trans*-stereoselectivity was observed, whereas when using EtAlCl_2_, MeAlCl_2_ and Me_2_AlCl_2_ the diastereoselectivity is favored to the formation of the *cis-*isomers.

**Scheme 32:**
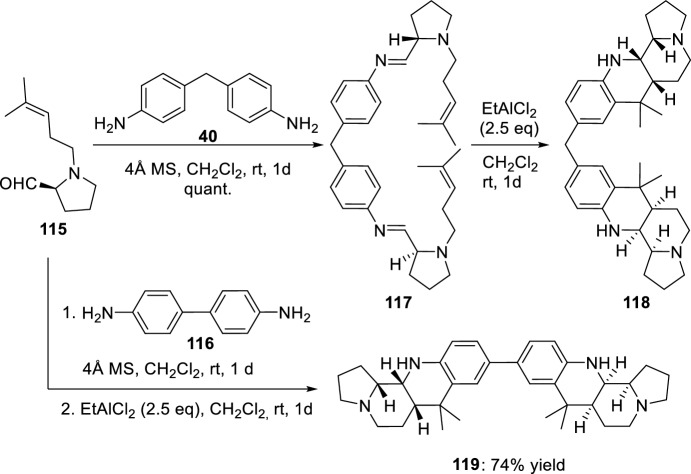
Synthesis of fused bis-benzopyrrolo[1,7]naphthyridine derivatives **118** and **119**

Laschat’s group studied the Povarov reaction of proline-derived aldehydes **115** with aromatic diamines **40** or **116** (Scheme 32) [52]. In this way, bis(benzo[*b*]pyrrolo[1,2-*h*][1,7]naphthyridine)methane **118** or **119** were isolated, respectively.

**Scheme 33:**
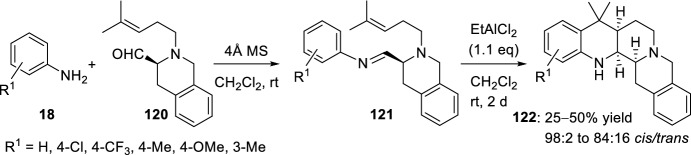
Synthesis of fused benzo[*g*]quinolino[2,3-*a*]quinolidines **122**

The same group studied the Lewis acid-catalyzed cyclization of *N*-aryl imines **121** obtained from l-phenylalanine derived aldehyde **120**, catalyzed by EtAlC1_2_ (Scheme 33) [53]. In this way, the benzo[*g*]quinolino[2,3-*a*]quinolidines **122** were obtained. The starting aldehyde **120**, prepared from l-phenylalanine, was treated with various aryl amines **18** in the presence of molecular sieves, giving rise to the corresponding imines **121**, which were immediately cyclized in the presence of EtAlC1_2_ to the benzo[*g*]quinolino[2,3-*a*]quinolidines **122**. The formal hetero-Diels–Alder reaction of **121** proceeded with high diastereoselectivity in favor of the *cis* configured product. The amino-substituent into a rigid pentacyclic system like **122** resulted in a good cytotoxic activity against human brain tumor cell lines.

**Scheme 34:**
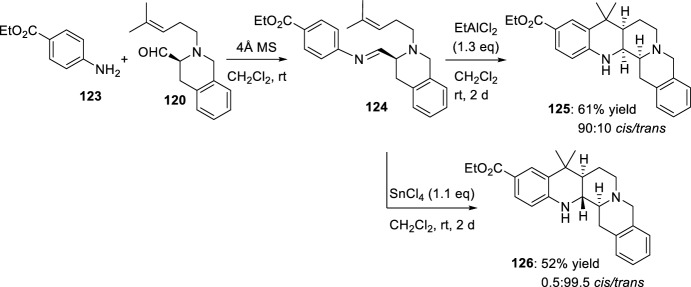
Synthesis of fused benzo[*b*]isoquinolino[2,3-*h*][1,7]naphthyridines **125** and **126**

On the one hand, the corresponding imine intermediate, obtained by condensation of aldehyde **120** with ethyl 4-aminobenzoate **123** in the presence of molecular sieves, directly treated with EtAlCl_2_, afforded the pentacyclic benzo[*b*]isoquino[2,3-*h*][1,7]naphthyridine **125** with high *cis*-diastereoselectivity (Scheme 34). Whereas, when the same imine was treated with SnCl_4_ the pentacyclic *trans*-diastereoisomer **126** was obtained in a dr 0.5/99.5 [54]. The authors highlight the interest of preparing polycyclic all-*trans-*derivatives because of their more planar shape, which could induce a different interaction mode with DNA.

**Scheme 35:**
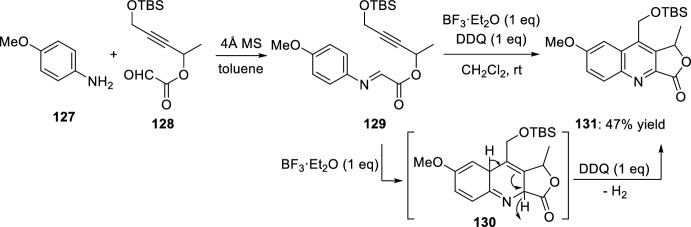
Synthesis of quinoline-fused lactones **131**

More aromatized fused heterocyclic compounds can be prepared directly by intramolecular Povarov reaction with triple-bond-functionalized aldehydes. When aldimines **129** derived from condensation of aromatic amines **127** with glyoxal-derived alkynes **128** are used, only one equivalent of DDQ is required to carry out the oxidation of dihydroquinoline-fused lactones **130**, obtained by intramolecular Povarov cycloaddition using 1 equivalent of BF_3_·OEt_2_ in CH_2_Cl_2_ at room temperature, affording the corresponding quinoline-fused lactones **131** (Scheme 35) [43].

**Scheme 36:**
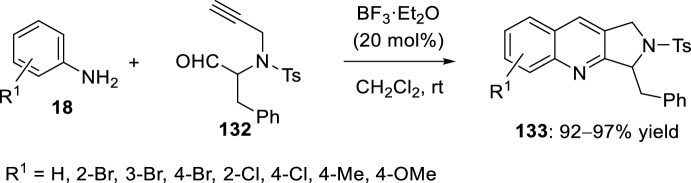
Synthesis of pyrrolo[3,4-*b*]quinolines **133**

Pyrrolo[3,4-*b*]quinolines **133** can also be synthesized by using propargyl aldehydes derived from α-amino acids **132** and various substituted anilines **18**. The intramolecular Povarov reaction requires a strategically positioned aldehyde moiety tethered to an alkynyl group. Hence, the reaction of *N*-propargyl aldehyde **132** and aromatic amines **18** was carried out in the presence of BF_3_·OEt_2_ in dry CH_2_Cl_2_. Using these reaction conditions, a series of pyrrolo[3,4-*b*]quinolines **133** were obtained in excellent yields (Scheme 36) [55].

**Scheme 37:**
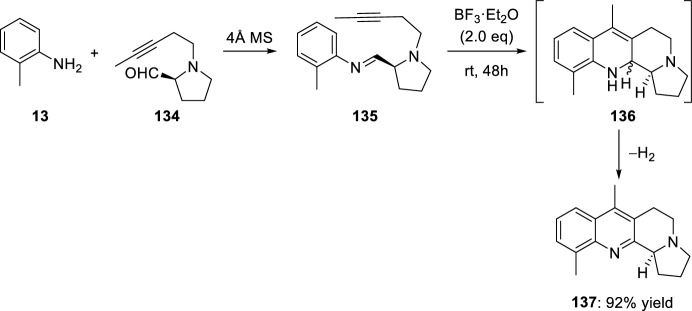
Synthesis of benzo[*b*]pyrrolo[1,2-*h*][1,7]naphthyridine derivative **137**

When l-proline-derived aldehyde **134** with tethered triple bond was used, benzo[*b*]pyrrolo[1,2-*h*][1,7]naphthyridine **137** was obtained (Scheme 37) [51]. Treatment of imine **135** bearing an internal alkyne moiety with BF_3_·OEt_2_ resulted in the clean formation of the indolizino[3,4-*b*]quinoline **137**. Obviously, the initially formed cyclization product **136** undergoes a rapid dehydrogenation to the aromatic compound **137**.

**Scheme 38:**
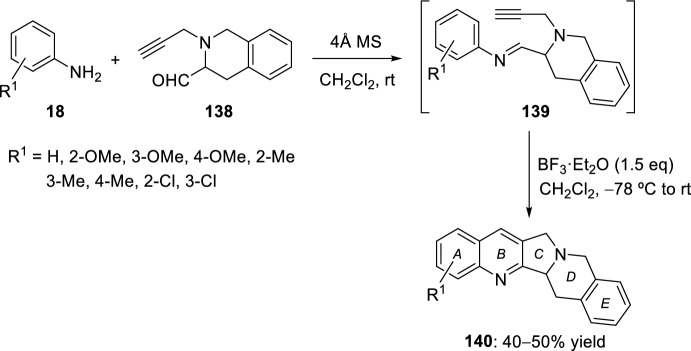
Synthesis of luotonin A analogs via intramolecular Povarov reaction

A small library of A- and D-ring modified luotonin-inspired heterocyclic systems was synthesized in moderate to good yields following a six-step route that starts from phenylalanine. The key step of this total synthesis consists in an intramolecular Povarov reaction of imines obtained from a tetrahydroquinoline-derived alkynyl aldehyde **138** and various aryl amines **18** (Scheme 38). The corresponding *N*-aryl imines **139** were formed in situ from aldehyde **138** and substituted aryl amines **18** in the presence of 4 Å molecular sieves. Without isolation, subsequent treatment of *N*-aryl imines **139** with 1.5 equivalents of BF_3_·OEt_2_ afforded the target pentacyclic heterocycles **140** in yields that were approximately in the 40–50% range [56].

**Scheme 39:**
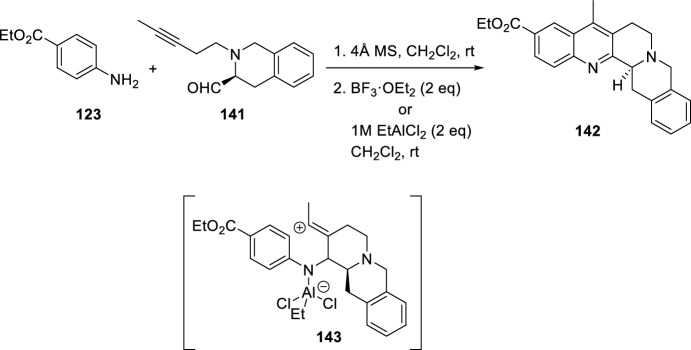
Synthesis of fused benzo[*b*]isoquinolino[2,3-*h*][1,7]naphthyridines **142**

When longer side chain with a triple bond is used in aldehyde **141**, benzo[*b*]isoquinolino[2,3-*h*][1,7]naphthyridine **142** is obtained (Scheme 39) [54]. In this case, aldehyde **141** was treated with ethyl 4-aminobenzoate **123** and the formation of the corresponding imine was observed and used further without purification. Subsequent addition of BF_3_·OEt_2_ and aqueous workup resulted in the formation of the quinoline ester **142** in 38% yield. When BF_3_·OEt_2_ was replaced by EtAlCl_2_, a chloro compound was isolated in 7% yield as a minor by-product. The isolation of this latter compound, further supports a cationic cyclization mechanism where in the presence of EtAlCl_2_ the cyclization of imine should afford carbenium ion **143**, which can undergo Friedel–Crafts-type electrophilic aromatic substitution, followed by tautomerization to give **142** after oxidation.

## Aromatic Amines and Aromatic Aldehydes

In this section, we disclose the intramolecular Povarov reaction using aromatic amines and aromatic aldehydes which allows the preparation of a diversity of polycyclic nitrogen containing heterocycles. Reactions have been classified considering the structure of the *ortho*-formylarenes and the way that the dienophile is tethered to the benzene ring.

### *C*-Alkenyl(Alkynyl) *Ortho*-Formylarenes

**Scheme 40:**
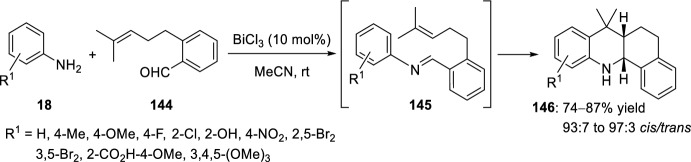
2-Prenylated benzaldehyde as carbonyl component in the BiCl_3_-promoted intramolecular Povarov reaction

Hexahydrobenzoacridine derivatives can be synthesized by intramolecular Povarov reaction of aldimines **145** derived from aromatic amines **18** and 2-prenylated benzaldehyde **144** (Scheme 40) [57]. The use of amines bearing electron-withdrawing or electron-donating groups in this approach, which was promoted by catalytic amount of bismuth(III) chloride, seems to have no effect on the reaction time or the yield. *cis*-Annulated hexahydrobenzo[*c*]acridines **146** were achieved in all cases with selectivities up to 97:3.

**Scheme 41:**
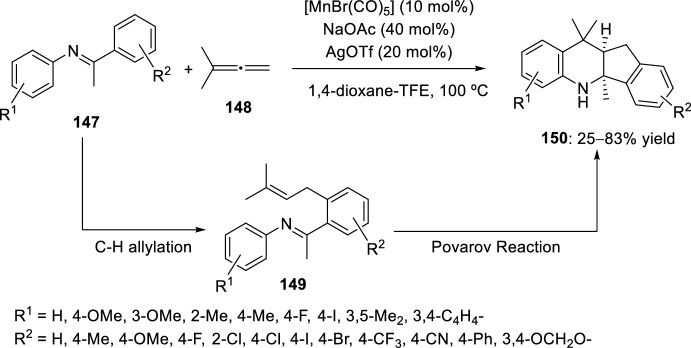
Synthesis of polycyclic compounds **150** through previous allylation followed by intramolecular Povarov reaction

Through a tandem allylation/intramolecular Povarov reaction, polycyclic compounds **150** were synthesized by a [4 + 2] cycloaddition process (Scheme 41). The imine group in compound **147** acts as a directing group to enable the introduction of a pendant alkene, thereby enabling a Lewis acid-catalyzed intramolecular Povarov reaction. Specifically, a manganese (I) complex catalyzed the directed C–H allylation with allene **148**, producing compound **149** ready for an in situ Povarov cyclization catalyzed by silver (I). Other Lewis acid, including BiCl_3_, Sc(OTf)_2_ and Zn(OTf)_2_, led to significant decomposition of the ketimine **149**. The reaction proceeds with high bond-forming efficiency (three C–C bonds), broad substrate scope, high regio- and *trans*-stereoselectivity, and 100% atom economy (Scheme 41). The polycyclic indenoquinoline, bearing two stereogenic centers, was obtained as a single diastereoisomer. The compatibility of different allenes was also examined, being the symmetric 1,1-dialkyl-substituted allenes the most efficiently coupling partners. The potential synthetic utility was demonstrated by a gram-scale synthesis [58].

**Scheme 42:**
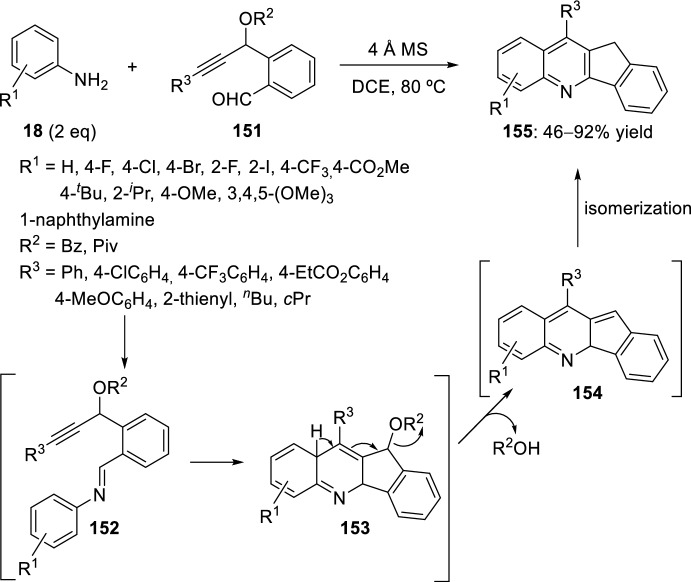
Synthesis of indeno[1,2-*b*]quinolines through reaction of aromatic amines and *o*-propargylbenzaldehydes

Liu et al. reported in 2013 the synthesis of indeno[1,2-*b*]quinolines **155** by means of reaction of aromatic amines **18** with *o*-propargylbenzaldehydes **151** (Scheme 42) [59]. Using a water-removing agent such as 4 Å molecular sieves compounds **155** were obtained in 1,2-dichloroethane (DCE) at 80 °C with 2 equivalents of functionalized aniline **18**. By reducing the amount of aromatic amine **18** to 1 equivalent, the yield of indenoquinoline **155** was affected by a significant decrease. A widespread diversity of substituted *o*-propargylbenzaldehydes **151** and aromatic amines **18** were appropriate for this transformation, giving to the formation of indeno[1,2-*b*]quinoline derivatives **155** in good to high yields. The mechanism of the formation of indenoquinolines **155** can start via initial formation of imine **152** by condensation of aromatic amines **18** with aldehydes **151**. The intramolecular Povarov reaction between azadiene moiety and alkyne group of **152** affords intermediates **153**. Elimination of OR^2^ group and subsequent double-bond isomerization furnish indenoquinolines **155** (Scheme 42).

**Scheme 43:**
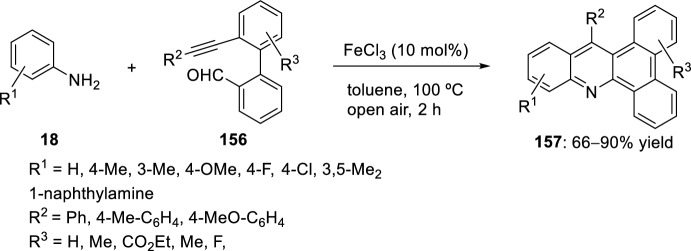
Synthesis of dibenzo[*a*,*c*]acridines using FeCl_3_

A modified Povarov reaction involving 2′-alkynylbiaryl-2-carbaldehydes **156** and aryl amines **18** with tandem oxidation was performed using catalytic FeCl_3_. The outcome was an efficient general synthesis of dibenzo[*a*,*c*]acridines **157** with moderate to high yields (Scheme 43). This method offers simplicity in the preparation of substrates, diverse substrate scope, and high atom economy. The optimum reactions conditions for the general synthesis of dibenzo[*a*,*c*]acridine derivatives **157** were obtained with a 10 mol% FeCl_3_ in toluene at 100 °C in open air. The synthesized compounds **157** had significant absorption and emission properties [60].

### *O*-Alkenyl(Alkynyl) *Ortho*-Formylarenes

**Scheme 44:**
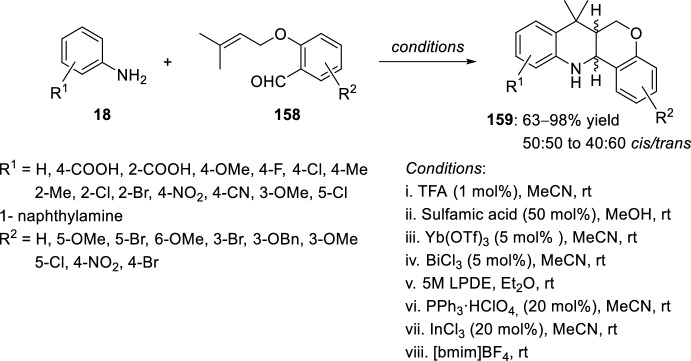
Synthesis of polysubstituted tetrahydrochromeno[4,3-*b*]quinolines **159** using different catalytic conditions

The reaction of aniline derivatives **18** with *O*-allyl derived salicylaldehydes **158** has been widely used for the synthesis of polysubstituted tetrahydrochromeno[4,3-*b*]quinolines **159** (Scheme 44). The intramolecular [4 + 2] cycloaddition reaction has been catalyzed in the presence of different Brønsted acids, such as trifluoroacetic acid (TFA) [61] and sulfamic acid [62], or in the presence of Lewis acids such as Yb(OTf)_3_ [61], BiCl_3_, [63] lithium perchlorate in diethyl ether (LPDE) [64], triphenylphosphonium perchlorate (TPP) [65], and InCl_3_ [66], and even in the presence of a recyclable ionic liquid as a reaction medium, [bmim]BF_4_ [67]. The reactions transcurred from good to excellent yields and a mixture of *cis*/*trans*-diastereoisomers was obtained in all cases.

**Scheme 45:**
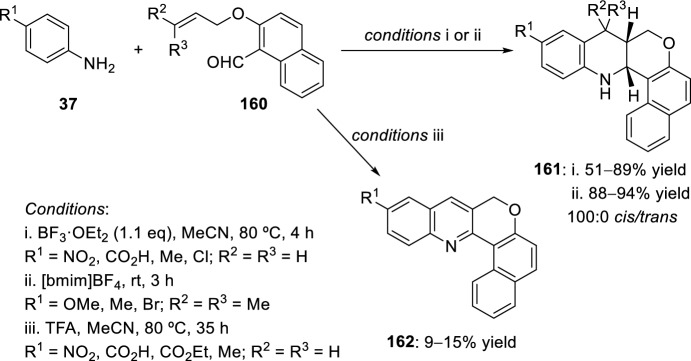
Synthesis of benzochromeno[4,3-*b*]quinoline derivatives **161** and **162**

Alternatively, condensation of 2-allyloxynaphthalene-1-carbaldehyde **160** with substituted anilines **37** and subsequent intramolecular cyclization in the presence of BF_3_·OEt_2_ yielded benzochromeno[4,3-*b*]quinolines **161** (Scheme 45) [68]. When the reaction was carried out with TFA, the obtained products were not those expected but their dehydrogenated derivatives **162**. In a similar manner, *cis*-compounds **161** could also be obtained performing the intramolecular aza-Diels–Alder reaction in [bmim]BF_4_ ionic medium [67], being the last one a green protocol that offers significant advantages over reported methods.

**Scheme 46:**
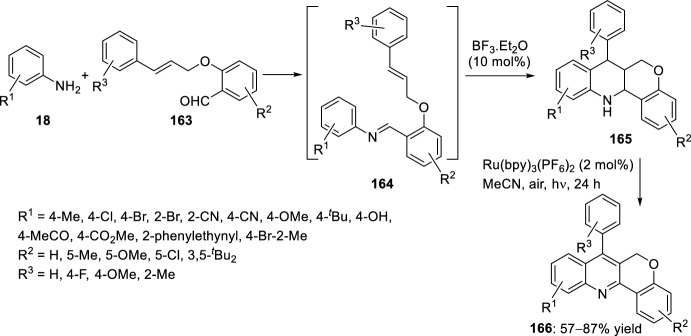
Preparation of chromeno[4,3-*b*]quinolines via intramolecular aza-Diels–Alder reaction

Substituted chromeno[4,3-*b*]quinolines **166** were achieved, under mild conditions, by tandem intramolecular aza-Diels–Alder reaction/photooxidation using a strategy of combination of visible-light photoredox and Lewis-acid catalysis. This intramolecular aza-Diels–Alder cycloaddition took place between the in situ generated benzylidene imine **164**, derived from aryl amines **18** and salicylaldehydes **163** bearing an alkene-tethered partner, followed by oxidative aromatization to give the products **166** (Scheme 46). The reaction takes place using BF_3_·OEt_2_ as Lewis acid and Ru(bpy)_3_(PF_6_)_2_ as photosensitizer in acetonitrile under aerobic condition with the irradiation of visible light [69].

**Scheme 47:**
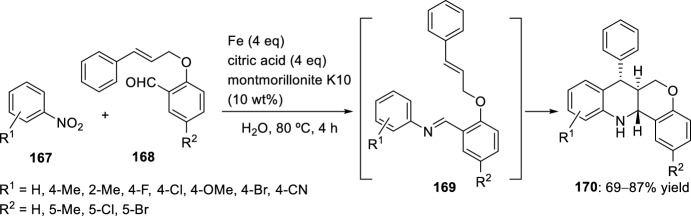
Synthesis of *trans*-fused tetrahydrochromeno[4,3-*b*]quinolines from nitrobenzenes **167**

The reaction between nitrobenzenes **167** and ω-unsaturated aldehydes, that is, 2-(cinnamyloxy)benzaldehydes **168**, in the presence of iron as reductant and catalytic amounts of montmorillonite K10 in aqueous citric acid at 80 °C produced *trans*-fused tetrahydrochromeno[4,3-*b*]quinolines **170** exclusively with yields ranging from 69% to 87% (Scheme 47) [70]. It is assumed that the sequence of reactions starts with an iron-mediated reduction of the nitrobenzene **167**. The resulting aniline reacts with the ω-unsaturated aldehyde **168** to give the corresponding imine **169**, which in turn undergoes an intramolecular aza-Diels–Alder reaction. This Povarov-type reaction is catalyzed by montmorillonite K10, proceeds via an *exo-*transition state structure, and delivers the *trans*-fused tetrahydrochromeno[4,3-*b*]quinolines **170** in diastereomerically pure form. The method enables the replacement of anilines with nitrobenzenes as substrates for intramolecular aza-Diels–Alder reactions. The domino reaction can be performed with numerous functionalized nitrobenzenes and a number of 2-(cinnamyloxy)benzaldehydes.

**Scheme 48:**
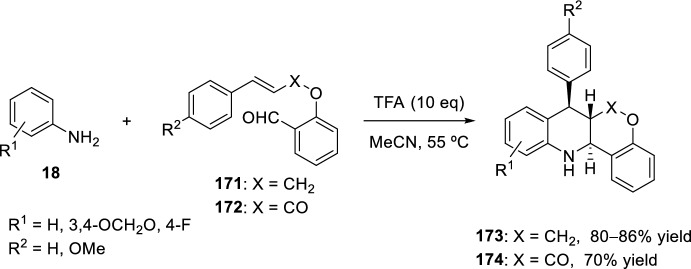
Intramolecular aza-Diels–Alder cyclization using *O*-cinnamyl- and *O*-cinnamoylsalicylaldehydes

Other configurational and functionally diverse heterocyclic compounds have also been prepared through an intramolecular formal aza-Diels–Alder cyclization. The substrates included substituted functionalized salicylaldehydes with a variety of anilines to yield different tetrahydroquinoline products [44]. Thus, cyclization of cinnamyl salicylaldehyde ethers **171** with substituted anilines **18** and treatment with trifluoroacetic acid in acetonitrile at 55 °C for 30 min afford the tetrahydroquinoline cycloadducts **173** in good yield (Scheme 48). Modest variations are well tolerated on the aniline ring, and the process can be extended to an electron-rich cinnamate ester **172** as well, although products **174** were obtained in modest yield. The major products isolated as single isomers after chromatographic or crystallographic purification possess the thermodynamically favored *trans*-configuration.

**Scheme 49:**
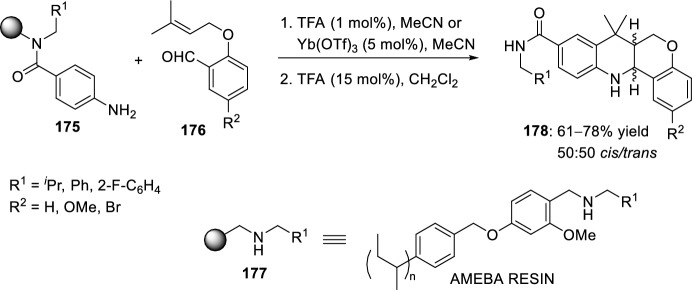
Solid-phase preparation of polysubstituted tetrahydrochromeno[4,3-*b*]quinolines **178** extended to solid phase

This methodology has also been extended to solid-phase synthesis with acid-sensitive methoxy benzaldehyde polystyrene (AMEBA) resin **177** (Scheme 49). The reaction of immobilized anilines **175** with salicylic aldehyde derivatives **176** containing an electron-rich olefin substituent, catalyzed by both TFA and Yb(OTf)_3_ yielded polysubstituted tetrahydrochromeno[4,3-*b*]quinolines **178** as 50:50 mixtures of diastereoisomers, which were subsequently separated by preparative HPLC [71].

**Scheme 50:**
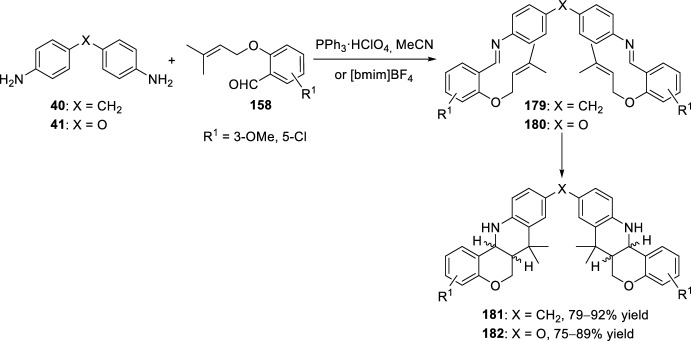
Preparation of bis-tetrahydrochromeno[4,3-*b*]quinolines using different reaction conditions

In addition, when using diamines and TPP or alternatively [bmim]BF_4_ as catalysts *bis*-tetrahydrochromeno[4,3-*b*]quinolines **181** or **182** could also be obtained (Scheme 50). The reaction of imines **179** or **180** derived from *O*-allyl salicylaldehydes **158** and 4,4′-methylenedianiline **40** or 4,4′-oxadianiline **41** over anhydrous Na_2_SO_4_ in acetonitrile and in the presence of 40 mol% of TPP, underwent intramolecular bis-cyclization to give the corresponding bis-4,4′-methylene **181** or 4,4′-oxatetrahydrochromeno[4,3-*b*]quinolines **182**, respectively, in good yields as a mixture of three isomers *cis*/*cis*, *cis*/*trans*, and *trans*/*trans* in a ratio of 1:1:1 (Scheme 50). The product ratio was determined by examination of the ^1^H-NMR spectrum of the crude product mixture [65]. Similarly, treatment of 4,4′-methylenedianiline **40** (X = CH_2_) with the *O*-prenyl derivative of salicylaldehyde **158** in [bmim]BF_4_ afforded the biscyclization product as a mixture of *cis*/*cis*, *cis*/*trans*, and *trans*/*trans*-isomers. However, in the case of 4,4′-oxadianiline **41** (X = O), the product was obtained exclusively as *cis*/*trans-*bis-adduct **182** under similar conditions [67].

**Scheme 51:**
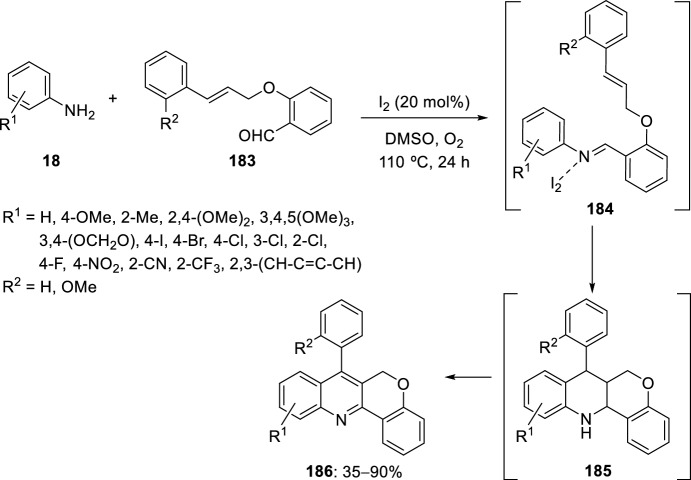
Synthesis of chromeno[4,3-*b*]quinolines promoted by I_2_/DMSO system

Very recently, Kouznetsov et al. [72] have described the synthesis of chromeno[4,3-*b*]quinolines **186** promoted by I_2_ and DMSO from easily available aryl amines **18** and *O*-cynnamyl salicyladehydes **183**. Iodine acts as a Lewis acid to catalyze the formation and cyclization of the imines **184**, using DMSO as the solvent, to generate the respective tetrahydrochromenoquinolines **185** as intermediates (Scheme 51). Finally, the I_2_/DMSO catalytic system could mediate the aromatization of **185** to the corresponding chromeno[4,3-*b*]quinolines **186**. The scope and general applicability of the reaction has been widely studied, considering both anilines and 2-(cinnamyloxy)benzaldehydes. The reaction proceeds under mild conditions, tolerates a great range of functional groups and features high step economy, since it constitutes a tandem process.

**Scheme 52:**
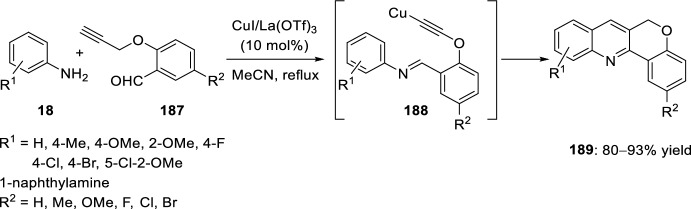
Efficient synthesis of 6*H*-chromeno[4,3-*b*]quinolines through CuI/La(OTf)_3_ promoted intramolecular Povarov reaction

Other strategies have been used for the synthesis of chromenoquinoline derivatives involving the use of *O*-propargyl-substituted salicylaldehyde ethers. In this context, activation of a terminal alkyne C–H bond by transition-metal catalysts is one of the major interests in synthetic organic chemistry. Several reports describe this activation by transition metal catalyst such as Ag, Au(I), Au(III), Cu(I), Ru, and Ir [73–76]. In this way, Nagarajan’s group [77] described a straightforward approach to chromenoquinolines using a mixture of copper(I) iodide and lanthanum triflate as an efficient catalyst. Moreover, copper compounds are readily available, non-air-sensitive, nontoxic catalysts and inexpensive, compared with other transition metal catalysts. 6*H*-Chromeno[4,3-*b*]quinolines **189** can be attained in good yields by intramolecular Povarov reaction of the intermediate aldimine **188** derived from the reaction of aromatic amines **18** with *O*-propargylated salicyladehydes **187** (Scheme 52). The combination of Cu(I) species/Lewis or Brønsted acids resulted in excellent catalytic properties, since the use of only a Lewis acid such as InCl_3_, BF_3_·OEt_2_, or La(OTf)_3_; or the use of a copper species such as CuI, CuBr or CuCl, afforded chromenoquinolines **189** with worse chemical yields. Aromatic amines **18** with ring-activating groups in *ortho*-, *meta*-, or *para*-positions participated in this reaction, giving the expected products with remarkably comparable yields (Scheme 52). Conversely, aromatic amines **18** with electron-withdrawing groups (R^1^ = NO_2_, CO_2_R, CN) did not afford the expected chromenoquinolines **189**. Moreover, substitution at *O*-propargylated salicyladehyde ring seems not to affect the reaction.

**Scheme 53:**
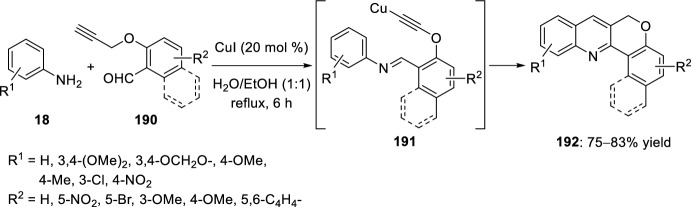
Copper-catalyzed intramolecular domino synthesis of 6*H*-chromeno[4,3-*b*]quinolines in green conditions

Alternatively, a green and simple intramolecular domino condensation aza-Diels–Alder reaction between anilines **18** and *O*-propargylated salicylaldehydes **190** in the presence of CuI as catalyst in H_2_O/EtOH was used to obtain 6*H*-chromeno[4,3-*b*]quinolines **192** in 75–83% yield (Scheme 53) [78]. A plausible mechanism assumes the formation of a copper-acetylide imine intermediates **191** that after the sequential intramolecular [4 + 2] cycloaddition, protonation, and oxidation generate the product **192**. The best yield was only obtained using highly electron-rich anilines. The simplicity of the starting materials, good yields of the products, and use of green, cheap, and nontoxic solvents are the main advantages of this method.

**Scheme 54:**
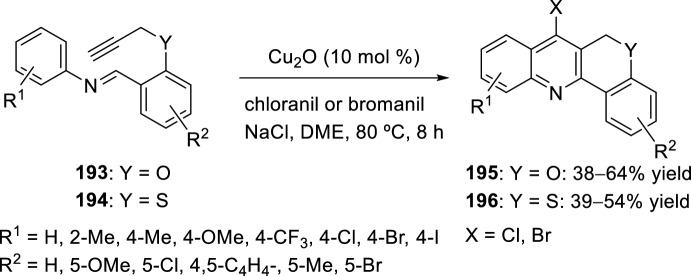
Synthesis of halogenated chromenoquinolines and thiochromenoquinolines via Cu-catalyzed cascade reaction

Complementarily, a new method was developed to synthesize 7-halogenated chromenoquinolines **195** and 7-halogenated thiochromenoquinolines **196**. The products can be directly obtained through Cu-catalyzed cascade reaction, that is, aza-Diels–Alder reaction of Schiff base **193** or **194**, followed by halogenation (chlorination or bromination), using chloranil or bromanil as halogen sources. Cu_2_O worked as both a Lewis acid and transition-metal catalyst in the aza-Diels–Alder reaction and halogenation reaction, respectively. Chloranil and bromanil also performed dual functions, that is, as a halogen source and oxidant. Although the halogenated products were obtained in moderate yields (Scheme 54), the present method is highly useful in organic synthesis because of mild reaction conditions and experimental simplicity [79].

**Scheme 55:**
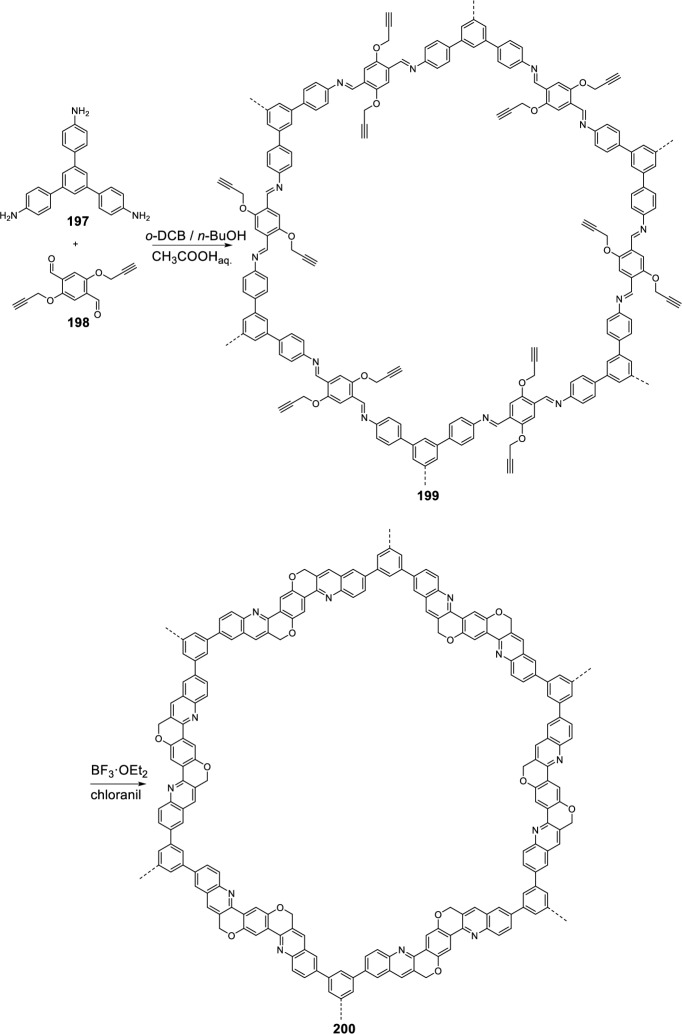
Synthesis of chromenoquinoline-COF_TAPB-BPTA_ **200**

**Fig. 2:**
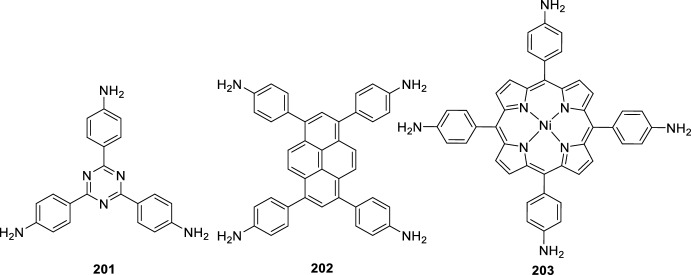
Other amines used to synthetize chromenoquinoline-based covalent organic frameworks (COF)

Very recently, Wang’s group [80] developed an intramolecular Povarov reaction for the construction of chemically stable chromenoquinoline-based covalent organic frameworks (COF). Thus, the synthesis involves the formation of the imine COF **199** by reaction of 2,5-bis-propargyloxy terephthalaldehyde (BPTA) **198** and 1,3,5-tris(4-aminophenyl)benzene (TAPB) **197** in a mixture of *o*-dichlorobenzene (*o*-DCB) and *n*-butanol (*n*-BuOH) followed by the addition of aqueous acetic acid (Scheme 55). Next, the intramolecular Povarov reaction to integrate the alkyne moieties into the imine COF **199** and to build the chromenoquinoline ring, was carried out using BF_3_·OEt_2_ as catalyst in toluene and in the presence of chloranil as an oxidating agent, leading the chromenoquinoline-COF_TAPB-BPTA_ **200** in a 96% yield (Scheme 55). Instead of TAPB **198** other amines, such as 1,3,6,8-tetrakis(4-aminophenyl)benzene **201**, 1,3,5-tris-(4-aminophenyl)triazine (TAPT) **202**, and Ni-porphyrin **203**, were also used to synthesize additional monomers with different symmetries and functional core moieties (Fig. 2). This novel approach achieves a high cyclization degree of 80–90%, which endows the chromenoquinoline-COFs with excellent chemical stability toward strong acid, base, and redox reagents. The absorption and fluorescence intensities of chromenoquinoline-COFs are sensitive to acid, which allows for dual-mode sensing of strongly acidic environments.

See Table 2 for the most representative examples of Sect. 3.2.

**Table 2:** Some examples of the intramolecular Povarov reaction between aromatic amines and *O*-alkenyl *ortho*-formylarenes

	
Entry	R^1^	R^2^	R^3^	R^4^	X	Catalyst	dr *cis/trans*	Yield (%)	References
1	H, 4-COOH, 2-CO_2_H, 4-OMe, 4-F, 4-Cl, 4-Me, 2-Me, 2-Cl, 2-Br, 4-NO_2_, 4-CN, 3-OMe, 5-Cl, 1-naphthylamine	H, 5-OMe, 5-Br, 6-OMe, 3-Br, 3-OBn, 3-OMe, 5-Cl, 4-NO_2_, 4-Br	Me	Me	CH_2_	TFA, sulfamic acid, Yb(OTf)_3_, BiCl_3_, 5 M LPDE, PPh_3_·HClO_4_, InCl_3_, [bmim]BF_4_	50:50 to 40:60	63–98	[61–67]
2	4-NO_2_, 4-CO_2_H, 4-Me, 4-Cl					BF_3_·OEt_2_	100:0	51–89	[68]
3	H, 4-OMe, 4-Me, 4-Br					[bmim]BF_4_	100:0	88–94	[67]
4	H, 3,4-OCH_2_O, 4-F	H	Ph, 4-MeOC_6_H_4_	H	CH_2_, CO	TFA	0:100	70–86	[44]
5	Solid-supported substituent in *p*-position	H, 5-OMe, 5-Br	Me	Me	CH_2_	TFA, Yb(OTf)_3_	50:50	61–78	[71]

### *N*-Alkenyl(Alkynyl) *Ortho*-Formylarenes

**Scheme 56:**
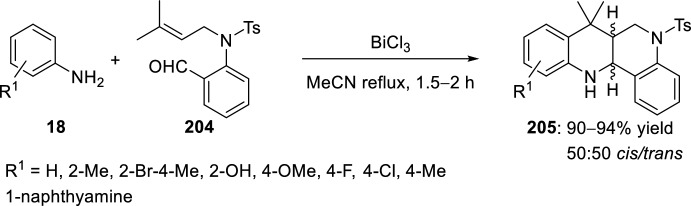
Preparation of hexahydrodibenzo[*b*,*h*][1,6]naphthyridines catalyzed by BiCl_3_ as Lewis acid

As in the case of *O*-allyl derivatives of salicylaldehydes, BiCl_3_ was used as Lewis acid to catalyze the intramolecular [4 + 2] cycloaddition reaction of in situ generated aldimines derived from aromatic amines and *o-*aminobenzaldehyde [81]. Therefore, treatment of anilines **18** with the *N*-allyl derivative of *o*-aminobenzaldehyde **204** in the presence of 10 mol% BiCl_3_ in refluxing acetonitrile resulted in the formation of hexahydrodibenzo[*b*,*h*][1,6]naphthyridines **205** as *trans*- and *cis*-diastereoisomers in a 1:1 ratio in excellent yields (Scheme 56).

**Scheme 57:**
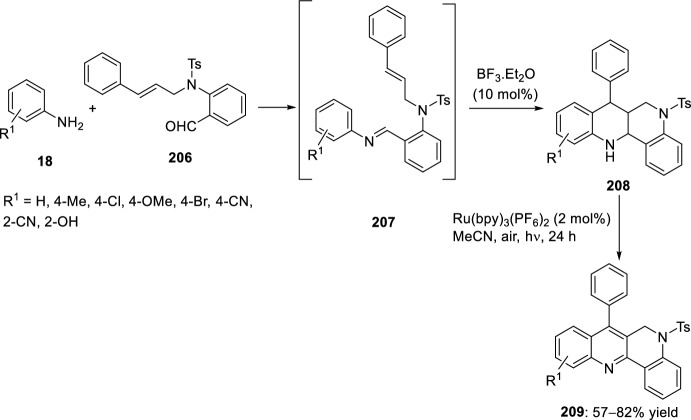
Preparation of 1,6-naphthyridines via tandem intramolecular aza-Diels–Alder reaction/oxidative aromatization

Alternatively, 1,6-naphthyridines **209** were achieved by tandem intramolecular aza-Diels–Alder reaction/oxidative aromatization using a strategy of combination of visible-light photoredox and Lewis acid catalysis. This intramolecular aza-Diels–Alder cycloaddition of the in situ generated benzylidene imines **207**, derived from aryl amines **18** and 2-aminoaryl aldehydes **206** bearing an alkene-tethered partner, took place followed by oxidative aromatization to give products **209** (Scheme 57). The reaction proceeds using BF_3_·OEt_2_ as Lewis acid and Ru(bpy)_3_(PF_6_)_2_ as photosensitizer in acetonitrile under aerobic condition with the irradiation of visible light. This method provided a new access to the synthesis of important heterocycles under mild conditions [69].

**Scheme 58:**
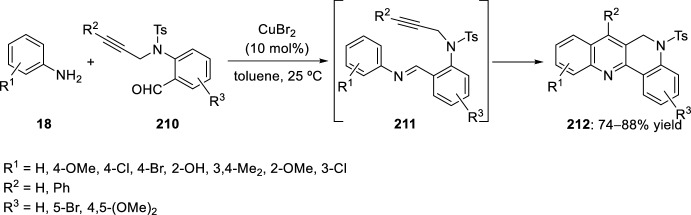
Synthesis of 5,6-dihydrodibenzo[*b*,*h*][1,6]naphthyridine derivatives via copper catalyzed reaction

Another highly efficient synthesis of 5,6-dihydrodibenzo[*b*,*h*][1,6]naphthyridines **212** was achieved by reaction between 2-(*N*-propargylamino)benzaldehydes **210** and aryl amines **18** in the presence of CuBr_2_ (Scheme 58). First, other copper halides were tested, such as CuCl, CuBr, and CuBr_2_, with the last one being the most efficient in terms of yield. Meanwhile, CuI and Cu(OAc)_2_ resulted to be inefficient. The proposed route involves that the in situ generated electron-deficient heterodienes **211** underwent an intramolecular inverse electron-demand hetero-Diels–Alder reaction followed by spontaneous dehydrogenation. This reaction tolerated a large number of substituents to afford diverse products under mild conditions [82].

## Aromatic Amines and Heteroaromatic Aldehydes

### Five-Membered Nitrogen-Containing Heterocyclic Alkene-Tethered Aldehydes

**Scheme 59:**
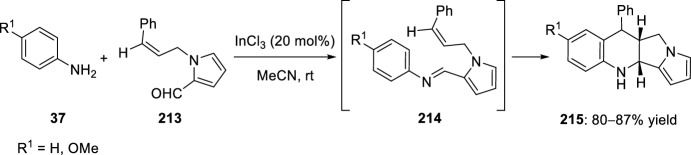
Synthesis of pyrrolizino[1,2-*b*]quinolines **215** by InCl_3_ promoted-intramolecular Povarov reaction

*N*-cinnamyl pyrrole-2-carbaldehyde **213** has been used as carbonyl component for the preparation of intermediate imines **214**, which readily cyclized in an intramolecular Povarov reaction to afford pyrrolizino-annulated quinoline derivatives **215** in good yields (Scheme 59) [83]. Indium trichloride proved to be an efficient Lewis acid catalyst for this transformation.

**Scheme 60:**
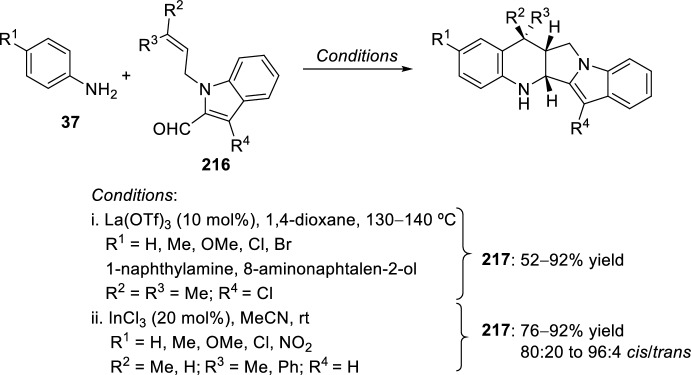
Lewis acid-catalyzed intramolecular Povarov reaction for the synthesis of indolo-annulated pyrroloquinolines

The synthesis of indolo-annulated pyrroloquinoline via the imino-Diels–Alder reaction has been described by Nagarajan et al. [84] In this case, Lewis acid-catalyzed intramolecular imino-Diels–Alder reaction of *N*-prenylated-2-formyl-3-chloroindole **216** (R^2^ = R^3^ = Me, R^4^ = Cl) and substituted anilines or naphthylamines **37** produced indolopyrroloquinolines **217** in moderate to excellent chemical yields and high *cis*-diastereoselectivity (Scheme 60). An array of Lewis acid catalyst has been tested in this approach and among them, La(OTf)_3_, Sc(OTf)_3_, and Yb(OTf)_3_ gave better diastereoselectivities. Only the *cis*-isomer was observed in the presence of La(OTf)_3_, when the reaction was performed at 130–140 °C. Similary, when indole-2-carbaldehydes containing an internal dienophile were used, indolo[2,1-*a*]pyrrolo[4′,3′:2,3]-7a,8,13,13b-tetrahydroquinolines **217** have been prepared from several substituted aromatic amines **37** through the intramolecular imino-Diels–Alder reaction (Scheme 60) [85]. *N*-alkenyl indole-2-carbaldehydes **216** (R^4^ = H) reacted with various *p*-substituted anilines **1** in the presence of different Lewis acid catalysts, namely AlCl_3_, BF_3_·OEt_2_, ZnCl_2_, and InCl_3_. However, the best overall yields were obtained when 20 mol% of InCl_3_ was used, and under these reaction conditions, the corresponding cycloadducts **217** were obtained in good overall yield and *cis*-diasteroselectivities ranging from 80:20 to 96:4.

**Scheme 61:**
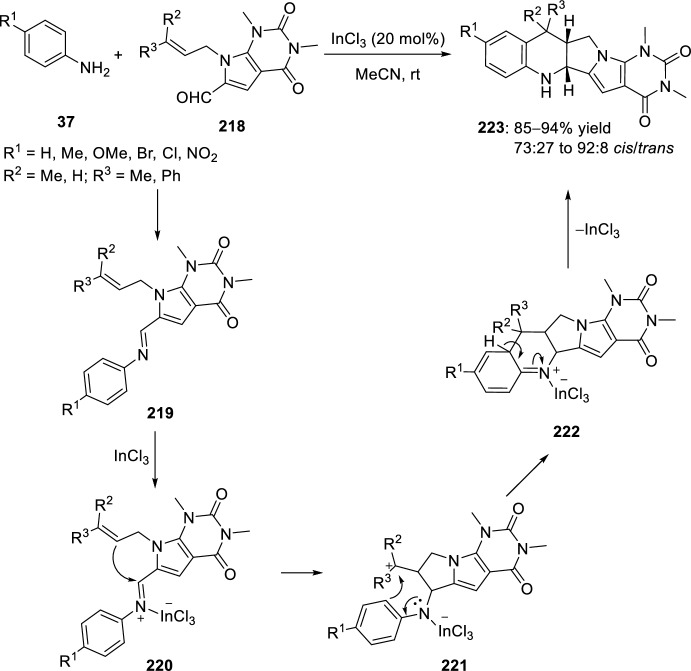
Synthesis of quinoline-annulated heterocycles by InCl_3_-assisted intramolecular Povarov reaction

*N*-alkenyl pyrrolopyrimidine-6-carbaldehydes **218** have also been used as carbonyl component for the preparation of intermediate imines, which readily cyclized in an intramolecular Povarov reaction to afford uracil-annulated quinoline derivatives **223** in good yields and good to excellent stereoselectivities (Scheme 61) [83, 86]. Different Lewis acid catalysts were studied in this transformation, namely BF_3_·OEt_2_, Yb(OTf)_3_, Sc(OTf)_3_, and InCl_3_. Indium trichloride proved to be the most efficient with overall yields higher compared with other tested Lewis acid catalysts [86]. These results indicate that the cyclization pathway proceeds by a stepwise mechanism as outlined in Scheme 61. Tetrahydroquinoline-annulated heterocycles **223** (R^1^ = Cl) were evaluated for their antibacterial activity against six different bacterial strains, being as active as the antibiotic ciprofloxacin and presenting a MIC value of 2.5 mg/mL against *Escherichia* *coli* [83].

**Scheme 62:**
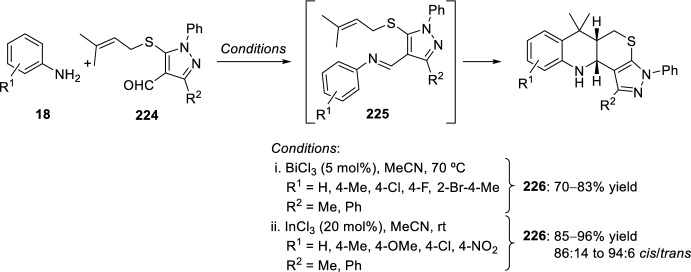
Pyrazole-annulated sulfur heterocycles by intramolecular Povarov reaction catalyzed by BiCl_3_ or InCl_3_

By using *N*-aryl imines **225**, generated in situ from anilines **18** and *S*-allyl-1*H*-pyrazole-4-carbaldehyde derivatives **224**, through an intramolecular imino-Diels–Alder reaction hexahydropyrazolo[4′,3′:5,6]thiopyrano[4,3-*b*]quinolines **226** have been prepared in good yields (Scheme 62) [87]. In this case, the reaction has been catalyzed by 5 mol% of BiCl_3_ and the process is highly diastereoselective by the exclusive isolation of the *cis*-cycloadduct. Some years later, Raghunathan et al. [88] reported similar synthesis of hexahydropyrazolo[4′,3′:5,6]thiopyrano[4,3-*b*]quinolines **226** by InCl_3_-promoted intramolecular Povarov reaction of *S*-allyl-1*H*-pyrazole-4-carbaldehyde derivatives **224** with substituted anilines **18** (Scheme 62). Cycloadducts **226** were obtained with 85–96% chemical yields and diastereoselectivities higher than 94:6 in favor of the *cis*-quinoline derivative.

**Scheme 63:**
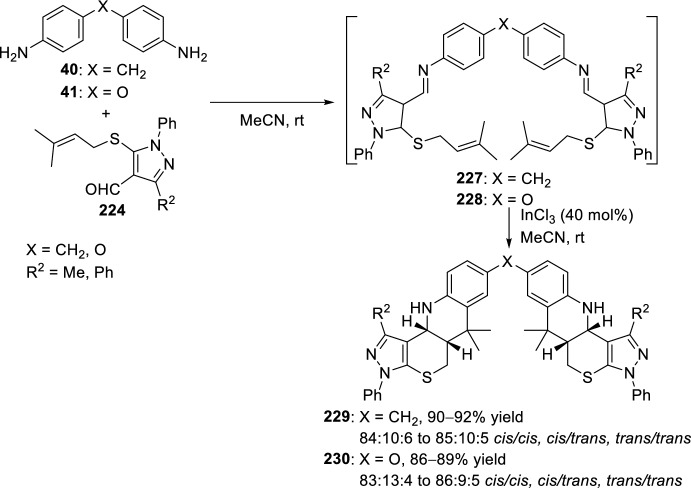
Pyrazole-annulated sulfur heterocycles by bis-intramolecular Povarov reaction catalyzed by InCl_3_

Reaction of aldehydes **224** and bis-aniline derivatives **40** or **41** affords intermediate aldimines **227** or **228**, which in the presence of 40 mol% of InCl_3_ undewent bis-intramolecular Povarov reaction to yield bis-tetrahydropyrazolo-thiopyrano[4,3-*b*]quinoline derivatives **229** or **230**, respectively, as a mixture of three inseparable isomers *cis*/*cis*, *cis*/*trans*, and *trans*/*trans* in favor of the *cis*/*cis*-isomer (Scheme 63) [88].

See Table 3 for the most representative examples of Sect. 4.1.

**Table 3:** Some examples of the intramolecular Povarov reaction between aromatic amines and five-membered nitrogen-containing heterocyclic alkene-tethered aldehydes

Entry		Aldehyde	Catalyst	Compound	dr *cis/trans*	Yield (%)	References
1	H, 4-OMe		InCl_3_		nr	80–87	[83]
2	H, 4-Me, 4-MeO, 4-Cl, 4-Br, 1-naphthylamine, 8-aminonaphtalen-2-ol		La(OTf)_3_		100:0	52–92	[84]
3	H, 4-Me, 4-MeO, 4-Cl, 4-NO_2_		InCl_3_		80:20 to 96:4	76–92	[85]
4	H, 4-Me, 4-MeO, 4-Br, 4-Cl, 4-NO_2_		InCl_3_		73:27 to 92:8	85–94	[83, 86]
5	H, 4-Me, 4-Cl, 4-F, 2-Br-4-Me		BiCl_3_		100:0	70–83	[87]
6	H, 4-Me, 4-MeO, 4-Cl, 4-NO_2_		InCl_3_		86:14 to 94:6	85–96	[88]

### Six-Membered Nitrogen- or Oxygen-Containing Heterocyclic Alkene-Tethered Aldehydes

**Scheme 64:**
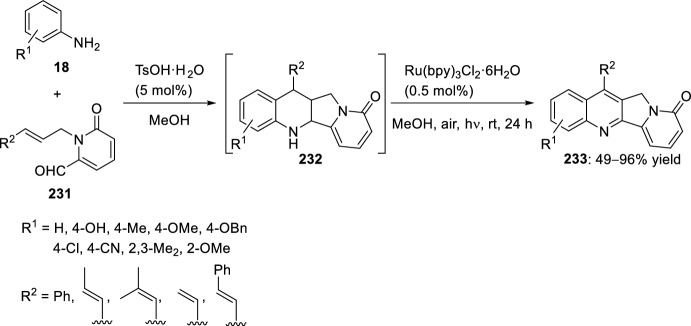
Combination of visible-light-photoredox and Brønsted acid-catalyzed intramolecular Povarov reaction for the preparation of indolizino[1,2‑*b*]quinolin-9(11*H*)‑ones

Recently, Zhang’s group [89] reported a one-step construction of substituted indolizino[1,2-*b*]quinolin-9(11*H*)-ones by combination of visible-light-photoredox and Brønsted acid catalysis through an intramolecular Povarov cycloaddition reaction under mild conditions. Thus, reaction of pyridine derivative-2-carbaldehyde **231** with anilines **18** in the presence of a photocatalyst and a Brønsted acid catalyst (TsOH) afforded indolizino[1,2-*b*]quinolin-9(11*H*)-ones **233** (Scheme 64). Both Ru(bpy)_3_Cl_2_·6H_2_O and Ru(bpy)_3_(PF_6_)_2_ were used as the photocatalyst, giving to the formation of tetracyclic compound **233** in more than 95% yield. Likewise, other acids, such as zinc trifluoromethanesulfonate (Zn(OTf)_2_), displayed a similarly high catalytic effectiveness. In this catalytic process, the visible-light-promoted dehydrogenation protocol of tetrahydroquinolines **232** constitutes the key procedure. The aniline substitution plays a crucial role in the success of the tetrahydroquinoline dehydrogenation step. Both weakly and strongly electron-donating groups (Me, OMe, OBn) at *para*-position of the aniline ring undergo excellent yields of compound **233**. Conversely, electron-withdrawing groups (Cl, CN) at this position of the aniline ring showed a negative effect on the reaction yield.

**Scheme 65:**
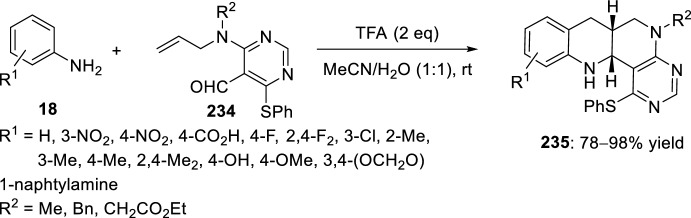
Intramolecular Povarov reaction in the preparation of benzopyrimido[4,5-*h*][1,6]naphthyridine libraries

A synthetic strategy developed in 2010 by Bai’s group [90] affords an efficient access to a series of libraries of the tetracyclic pyrimidine-fused heterocycles. The key step in this synthetic methodology entails the intramolecular Povarov reaction of imine intermediate formed in situ from the reaction of aromatic amines **18** and allylaminopyrimidine-5-carbaldehydes **234**. Trifluoroacetic acid was selected as Brønsted acid catalyst to accomplish this transformation, affording exclusively *cis*-benzopyrimido[4,5-*h*][1,6]naphthyridines **235** in good to excellent yields (Scheme 65). Although the use of 10 mol% TFA in acetonitrile yielded the desired product in good yields, increasing the catalyst loading to 2 equivalents led to shorter reaction rates and higher yields.

**Scheme 66:**
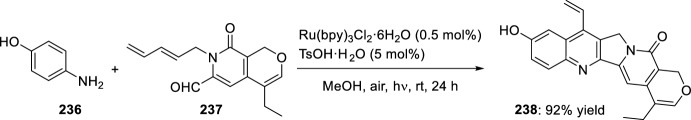
Tandem acid-catalyzed intramolecular Povarov reaction/visible-light photoredox for the synthesis of the precursor of 10-hydroxycamptothecin and irinotecan

The combination of visible-light-photoredox and acid catalysis has also been applied to the formal synthesis of the precursor of 10-hydroxycamptothecin and irinotecan. The intramolecular Povarov cycloaddition/dehydrogenation aromatization cascade of pyridone carbaldehyde **237** and 4-aminophenol **236** in the presence of a photocatalyst (Ru(bpy)_3_Cl_2_·6H_2_O) and a Brønsted acid catalyst *p*-toluenesulfonic acid (*p*-TsOH) yielded pentacyclic derivative **238** in 92% yield (Scheme 66) [89].

**Scheme 67:**
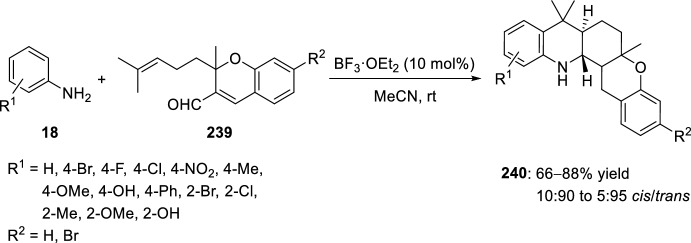
Synthesis of chromenoacridine derivatives through intramolecular Povarov reaction

In 2010 Subba Reddy et al. [91] described the first synthesis of pentacyclic polyaromatic chromenoacridine derivatives in a single-pot operation. This protocol involves the formation of the intermediate imine between *para*- and *ortho*-substituted aromatic amines **18** and alkene-tethered chromene-3-carbaldehyde **239**, followed by the BF_3_·OEt_2_-induced intramolecular Povarov reaction. Under these reaction conditions a set of 18 chromeno[2,3-*c*]acridines **240** were obtained in 66–88% yield with high *trans*-steroselectivity (Scheme 67). Other Lewis acids such as AlCl_3_, FeCl_3_, ZnCl_2_, SnCl_4_, Sc(OTf)_3_, InCl_3_, InBr_3_, In(OTf)_3_, LiClO_4_, and Brønsted acid TFA, were ineffective for this transformation in terms of both yield and selectivity. All attempts to extend this protocol to diamines such as 1,5-diaminonaphthalene did not furnish the desired product.

**Scheme 68:**
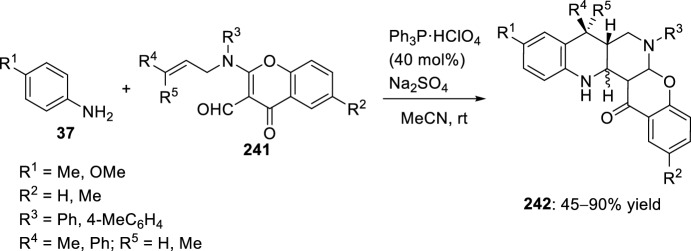
Synthesis of chromenonaphthyridines via TPP-induced intramolecular Povarov reaction

Alkene-tethered aminochromene-3-carbaldehyde **241** has been employed for the intramolecular inverse electron demand [4 + 2] cycloaddition reaction [92]. 2-(*N*-Alkenyl-*N*-aryl)aminochromene-3-carbaldehyde **241** also underwent intramolecular Povarov reaction with aromatic amines **37** in the presence of Lewis acids to furnish chromenonaphthyridines **242** (Scheme 68) [93]. Thus, reaction of *para*-substituted aromatic amines **37** with **241** in the presence of 40 mol% of PPh_3_·HClO_4_ (TPP)[65], afforded *cis*- or *trans*-chromenonaphthyridines **242**. *cis*-Adduct **242** is favored when R^4^ = Me, while *trans-*chromenonaphthyridines **242** was observed when R^4^ = Ph.

**Scheme 69:**
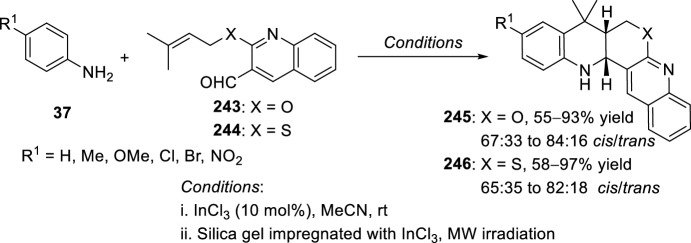
Synthesis of pyrano and thiopyranoquinolines through InCl_3_/silica gel supported catalyzed intramolecular Povarov reaction

Raghunathan et al. [94] reported in 2008 a simple procedure for the synthesis of pyrano and thiopyranoquinoline derivatives using indium trichloride supported in silica gel. *O*-alkenyl **243** and *S*-alkenylquinoline-3-carbaldehyde **244** are suitable starting materials to undergo intramolecular Povarov reaction with a variety of aromatic amines **37**. Therefore, reaction of aromatic amines **37** with **243** or **244** in the presence of InCl_3_ in acetonitrile furnished a mixture of *cis*- and *trans*-pyrano **245** and thiopyranoquinolines **246**, respectively, with diastereoselectivities ranging from 65:35 to 84:16 by intramolecular Povarov reaction of the intermediate imine generated in the one-pot reaction (Scheme 69). As a further extension of this work, the same reaction was carried out using InCl_3_ impregnated in silica gel as Lewis acid catalyst under microwave irradiation. These reaction conditions dramatically increase the overall yields from 55–82% to 75–97%, retaining nearly the same diastereoselectivity ratios. This ecofriendly protocol avoids the use of organic solvents, has general applicability, and notably enhances reaction rates and chemical yields.

**Scheme 70:**
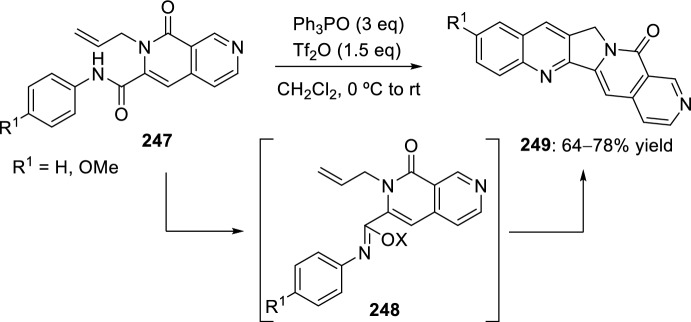
Cyclization of *N*-allyl naphthyridones into luotonin A analogs using amide-activating reagents

In 2008, Zhang’s group developed a new intramolecular Povarov reaction for the preparation of luotonin A analogs [95]. This approach entails the in situ formation of imidates through activation of corresponding chemically stable amides. Thus, bis(triphenyl)oxodiphosphonium trifluoromethanesulfonate, formed in situ from Ph_3_PO and Tf_2_O used for the total synthesis of camptothecin [96] and luotonin A [97], works as an amide-activating reagent to convert the amide moiety to its corresponding imidate under mild reaction conditions, and also to promote the subsequent intramolecular Povarov reaction in the desired direction. Using these catalytic conditions, cyclization of *N*-allyl naphthyridones **247** to afford luotonin A analogs **249** through the corresponding imidates **248** was attained in 64–78% yield (Scheme 70). The formation of compound **249** may be rationalized by the stability of aromatic system, driven by the catalytic system acidity.

**Scheme 71:**
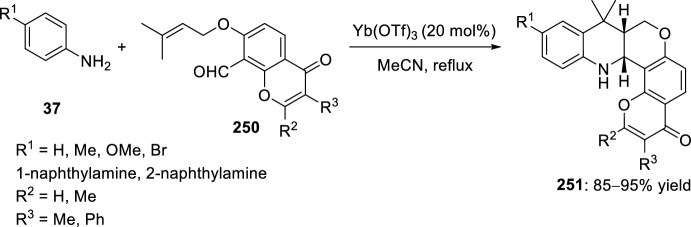
Povarov reaction in the preparation of antiproliferative tetrahydropyranochromeno[4,3-*b*]quinolines

Nagaiah et al. [98] reported the diastereoselective synthesis of tetrahydropyrano-chromeno[4,3-*b*]quinolines **251** by intramolecular Povarov reaction of formal 2-azadienes obtained in situ from aromatic amines **37** and *O*-prenyladed compounds **250** derived from 8-formyl chromenones (Scheme 71). Several Lewis and Brønsted acid catalysts were tested in this reaction and, among them, Yb(OTf)_3_ and Sc(OTf)_3_ were found to be almost equally efficient according to reaction yields, times, and diastereoselectivities. Independent of the nature of the catalyst, in all cases studied exclusive formation of *cis*-tetrahydrochromenoquinolines **251** was obtained, which may be due to the steric effect of the chromenone moiety. This method allows the use of aromatic amines **37** with electron-withdrawing or electron-donating groups, giving compounds **251** in very good yields. Some of these synthesized tetrahydrochromeno[4,3-*b*]quinolines **251** exhibited significant antiproliferative activity against MCF-7 breast cancer cell line and low inhibitory activity against MDA-MB-231 breast cancer cell line.

**Scheme 72:**
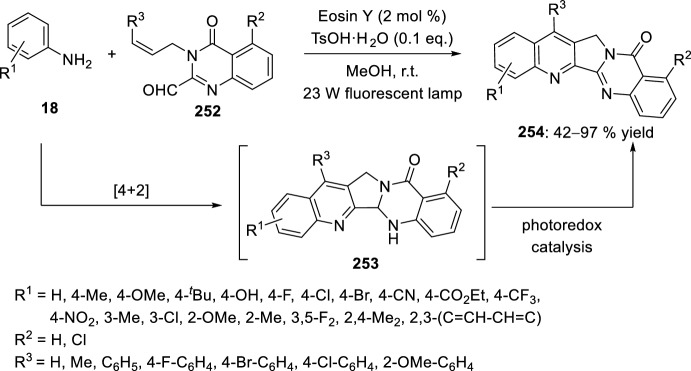
Tandem intramolecular Povarov reaction/visible light-promoted dehydrogenation for the construction of substituted luotonin A derivatives

Very recently, Zhang’s group [99], continuing their work on heterocyclic compound synthesis under visible light, has developed an intramolecular Povarov cycloaddition reaction to construct substituted luotonin A via visible-light-promoted dehydrogenation of pentacyclic pyrroloquinazolines **254** (Scheme 72). The optimized reaction conditions consisted in using eosin Y as the photocatalyst and TsOH·H_2_O as the co-catalyst in MeOH under the irradiation using a normal 23 W household lamp at room temperature. Thus, when 3-cynnamyl-4-oxo-3,4-dihydroquinazoline-2-carbaldehydes **252** reacted with anilines **18**, the pentacyclic pyrroloquinazolines **254** were obtained with up to 97% yield. Control experiments confirmed the necessity of both visible light and oxygen. In the absence of photocatalyst or acid the yield significantly decreased. At the same time, the reaction could not proceed in the dark or under a N_2_ atmosphere.

See Table 4 for the most representative examples of Sect. 4.2.

**Table 4:** Some examples of the intramolecular Povarov reaction between aromatic amines and six-membered nitrogen- or oxygen-containing heterocyclic alkene-tethered aldehydes

Entry		Heteroaromatic aldehyde	Catalyst	Compound	dr *cis/trans*	Yield (%)	References
1	H, 3-NO_2_, 4-NO_2_, 4-CO_2_H, 4-F, 2,4-F_2_, 3-Cl, 2-Me, 3-Me, 4-Me, 2,4-Me_2_, 4-OH, 4-OMe, 3,4-(OCH_2_O), 1-naphtylamine		TFA		100:0	78–98	[90]
2	H, 4-Br, 4-F, 4-Cl, 4-NO_2_, 4-Me, 4-OMe, 4-OH, 4-Ph, 2-Br, 2-Cl, 2-Me, 2-OMe, 2-OH		BF_3_·OEt_2_		10:90 to 5:95	66–88	[91]
3	4-Me, 4-OMe		Ph_3_P·HClO_4_		100:0	45–90	[93]
4	4-Me, 4-OMe		Ph_3_P·HClO_4_		0:100	58–77	[93]
5	H, 4-Me, 4-OMe, 4-Cl, 4-Br, 4-NO_2_		InCl_3_		67:33 to 84:16	55–93	[94]
6	H, 4-Me, 4-OMe, 4-Cl, 4-Br, 4-NO_2_		InCl_3_		65:35 to 82:18	58–97	[94]
7	H, 4-Me, 4-OMe, 4-Br, 1-naphthylamine, 2-naphthylamine		Yb(OTf)_3_		100:0	85–95	[98]

### Oxygen- or Nitrogen-Containing Heterocyclic Alkyne-Tethered Aldehydes

**Scheme 73:**
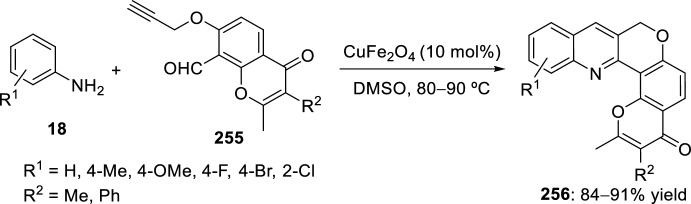
*O*-Propargylated 8-formyl chromenones as carbonyl components in the CuFe_2_O_4_-promoted intramolecular Povarov reaction

*O*-Propargylated compounds derived from 8-formyl chromenones **255** have been used as carbonyl compounds in the preparation of pyranochromeno[4,3-*b*]quinolines **256** [100]. Authors analyze the activity of several copper catalyst for the activation of the terminal alkyne C–H bond in **255** by the use of CuFe_2_O_4_ nanoparticles, CuI, Cu(OTf)_2_, CuCl, and CuBr. CuFe_2_O_4_ nanoparticles were found to be the best catalyst for this transformation, which due to their magnetic properties can be easily separated from the reaction mixture and reused without loss of activity. In addition, the choice of DMSO as the solvent among others (i.e. MeCN, toluene, DMF, H_2_O) was done in terms of reaction efficacy. Electron-donating groups at *ortho* or *para*-positions in aromatic amines **18** gave good yields of pyranochromenoquinolines **256** (Scheme 73). However, anilines with electron-withdrawing groups did not afford the desired adducts **256** when reacted with *O*-propargylated-8-formyl chromenones **255**.

**Scheme 74:**
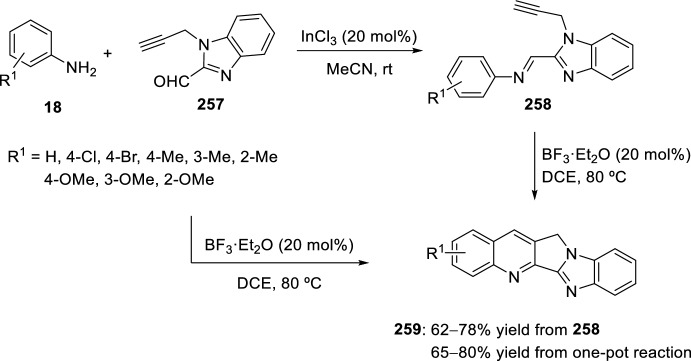
Synthesis of decarbonyl analogs of the anticancer alkaloid luotonin A by BF_3_·OEt_2_-assisted intramolecular Povarov reaction

The intramolecular Povarov reaction using *N*-containing heterocyclic *N*-alkyne-tethered aldehydes has been applied for the preparation of alkaloids with fused heterocycles. Menéndez’s group [101] reported in 2017 a small library of benzimidazole-fused pyrrolo[3,4-*b*]quinolines **259** synthesized from readily available benzimidazole 2-carbaldehyde **257** and various substituted aryl amines **18**. Under catalytic-free conditions or in the presence of InCl_3_, Yb(OTf)_3_, InBr_3_, or ammonium cerium(IV) nitrate (CAN), in different solvents such as MeCN, CH_2_Cl_2_, and 1,2-dichloroethane (DCE); the corresponding cycloadducts **259** derived from the intramolecular Povarov reaction were not obtained and only the corresponding aldimines **258** were attained instead (Scheme 74). However, treatment of aldimines **258** with 20 mol% of BF_3_·OEt_2_ in DCE at 80 °C afforded pyrrolo[3,4-*b*]quinolines **259** in good yields. In addition, cycloadducts **259** were achieved in 65–80% yield via one-pot intramolecular Povarov reaction when substituted anilines **18** reacted with benzimidazole 2-carbaldehyde **257** in the presence of BF_3_·OEt_2_. Compounds, thus synthesized, can be considered as decarbonyl analogs of the anticancer alkaloid luotonin A and were evaluated in a DNA relaxation assay for their ability to inhibit human topoisomerase I.

**Scheme 75:**
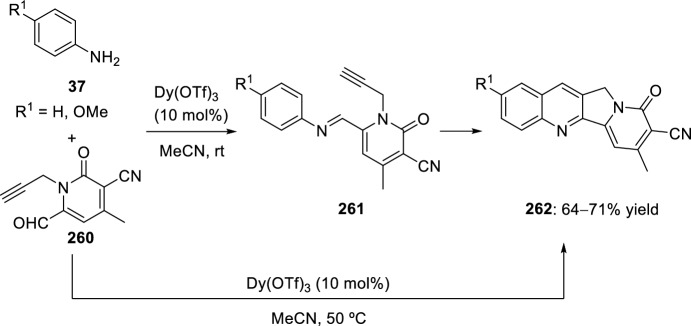
Intramolecular Povarov reaction in the formal synthesis of camptothecin

Batey’s group [102] reported the intramolecular Povarov reaction employing *N*-propargylic-substituted aldehydes **260** derived from pyridine for the synthesis of the pyrrolo[3,4-*b*]quinoline nucleus of camptothecin (Scheme 75). When aldehyde **260** and aniline reacted in the presence of 10 mol% of Dy(OTf)_3_ at room temperature, the corresponding imine **261** was isolated, whereas when the reaction was carried out at 50 °C quinoline **262** was directly obtained. The formation of quinoline derivative **262** (R^1^ = H) constitutes a formal synthesis of camptothecin, while the obtained compound **262** derived from *p*-anisidine **37** (R^1^ = MeO) can be used as precursor in the preparation of topotecan.

**Scheme 76:**
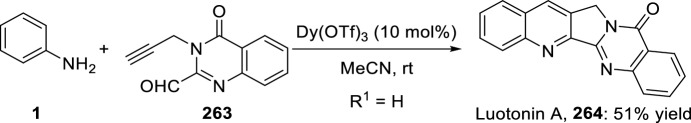
Intramolecular Povarov reaction in the total synthesis of luotonin A

Similarly, this methodology allowed the synthesis of luotonin A **264** in 51% yield from the intramolecular Povarov reaction of *N*-propargylic-substituted aldehyde derived from quinazoline **263** and aniline **1** in the presence of 10 mol% Dy(OTf)_3_ in acetonitrile (Scheme 76) [102].

**Scheme 77:**
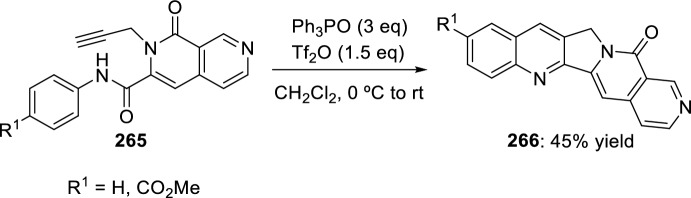
Cyclization of *N*-propargyl naphthyridones into luotonin A analogs using amide-activating reagents

Luotonin A analogs [95] have also been prepared by intramolecular Povarov reaction through in situ formation of imidates by activation of corresponding chemically stable amides. Bis(triphenyl)oxodiphosphonium trifluoromethanesulfonate, an amide-activating reagent, catalyzes the cyclization of *N*-propargyl naphthyridones **265** yielding luotonin A analogs **266** in moderate yield (Scheme 77).

## Heteroaromatic Amines and Aliphatic Aldehydes

**Scheme 78:**
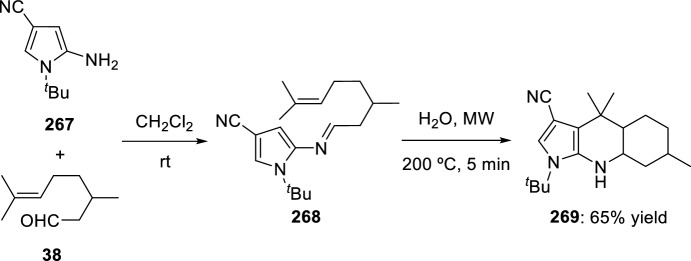
Intramolecular Povarov reaction using 2-aminopyrrole under microwave conditions

Amino-heterocycles have scarcely been exploited as building blocks for either inter- or intramolecular imino-Diels–Alder reaction, even though they can condense with aldehydes to form imine derivatives. As far as we know, only two examples have been reported in the intramolecular Povarov reaction of imines generated from heteroaromatic amines and aliphatic alkene-tethered aldehydes. For instance, a catalyst-free intramolecular imino-Diels–Alder protocol for the synthesis of annulated tetrahydropyridines has been developed by Vilches-Herrera et al. [103]. The corresponding octahydro-1*H*-pyrrolo[2,3-*b*]quinoline **269** was obtained in 65% yield via cycloaddition of aldimine **268** obtained by condensation reaction between 2-aminopyrrole **267** and a non-aromatic aldehyde such as citronellal **38** (Scheme 78). The reaction is carried out in water under microwave irradiation at 200 °C and with no catalyst, which fulfills all the requirements for sustainable chemistry.

**Scheme 79:**
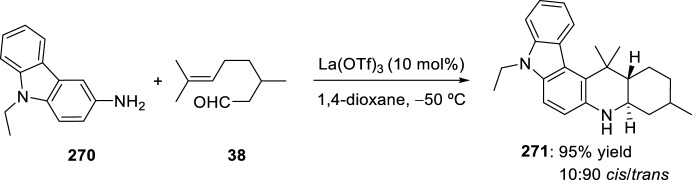
Povarov reaction of citronellal and 3-aminocarbazole

The Nagarajan’s group [104] reported in 2008 the synthesis of indoloacridine **271** in the intramolecular Povarov reaction of imine derived from aliphatic citronellal **38** as dienophile and a heteroaromatic amine such as 3-aminocarbazole **270** (Scheme 79). The reaction proceeded very smoothly in the presence of Lewis acid La(OTf)_3_. Although the diastereoselectivity is highly temperature dependent, the *trans*-isomer is the major product isolated in this reaction, and better diastereoselectivities (10:90) were attained at low reaction temperatures.

## Heteroaromatic Amines and Aromatic Aldehydes

**Scheme 80:**
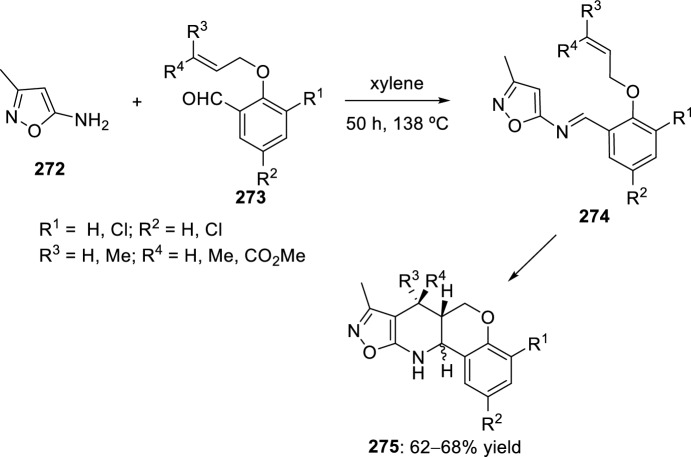
Hetero-Diels–Alder reaction of 5-amino-3-methylisoxazole in the synthesis of annulated tetrahydropyridines

The first example was reported by Tietze et. al. in 1992. They worked out the intramolecular Diels–Alder reaction between benzaldehydes **273** and aminoisoxazole **272** under thermal conditions (Scheme 80) [105]. In this case, the condensation of both reagents generated the corresponding imines **274**, which can be isolated, and selectively cyclized to form the *cis*- or *trans*-fused tetrahydropyridines **275** (Scheme 80). The selectivity of these reactions could be explained by electronic effects. In the reaction of **273** (R^1^ = R^2^ = R^3^ = R^4^ = H) with **272** only the *trans*-annulated tetrahydropyridine **275** was obtained. In addition, during the reaction of **272** with **273** (R^1^ = R^2^ = R^3^ = H, R^4^ = CO_2_Me) two diastereoisomers could be formed, but only the *trans*-annulated tetrahydropyridine **275** was observed. Surprisingly, the reaction of **273** (R^1^ = R^2^ = Cl, R^3^ = R^4^ = Me) with **272** yielded only the *cis*-fused compound **275**.

**Scheme 81:**
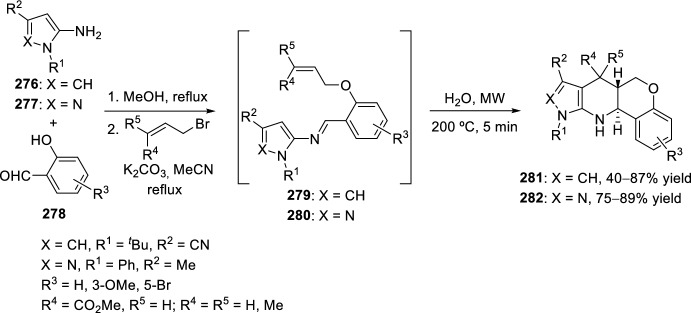
Intramolecular Povarov reaction using 2-aminopyrrole and 2-aminopyrazole under microwave conditions

A highly diastereoselective methodology for the aza-Diels–Alder cycloaddition using 2-aminopyrrole derivatives **276** (X = CH) or 2-aminopyrazole derivatives **277** (X = N) to construct chiral tetracyclic hexahydrochromenopyrrolo-pyridines **281** and hexahydrochromenopyrazolo-pyridines **282** was developed (Scheme 81) [103]. In a first step, imine derivatives **279** or **280** were previously synthesized through condensation of commercially available 2-hydroxybenzaldehyde derivatives **278** with **276** or **277** and subsequent alkylation reaction. The [4 + 2] cycloaddition reaction was conducted in water under microwave irradiation and with no catalyst (Scheme 81). Moreover, in most of the cases the products precipitate in the reaction media, avoiding the use of solvents for extraction and column chromatography for purification. The reaction is solvent dependent with regard to its stereoselectivity. Only the *trans*-isomer is obtained if the reaction is performed in water, whereas in a nonpolar solvent such as *p*-xylene the *cis*-isomer can also be isolated.

**Scheme 82:**
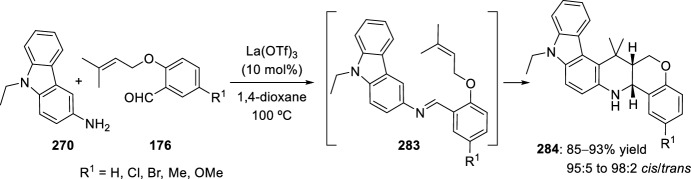
Intramolecular Povarov reaction of aromatic aldehydes and 3-aminocarbazole

As an extension of the methodology developed by Nagarajan’s group, which used 3-aminocarbazol **270** as heteroaromatic amine and La(OTf)_3_ as Lewis acid (vide supra Scheme 79), the reaction was also performed with imines **283** derived from aromatic aldehydes (*O*-prenylated salicylaldehydes) **176** [104]. Isomeric ellipticine derivatives **284** were obtained in 85–92% yield and very good diastereoselectivities (95:5–98:2) in favor of *cis*-isomer (Scheme 82).

**Scheme 83:**
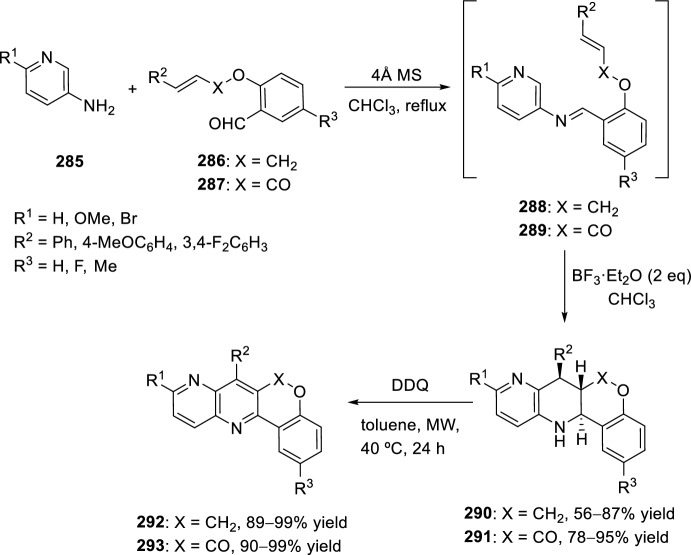
Synthesis of chromeno[4,3-*b*][1,5]naphthyridines and chromeno[4,3-*b*][1,5]naphthyridin-6-ones

Palacios et al. have described the synthesis of 1,5-naphthyridine derivatives fused with other oxygen-containing heterocycles such as chromenes or chromen-2-ones [106]. The synthetic route involves an intramolecular [4 + 2] cycloaddition reaction using BF_3_·OEt_2_ as Lewis acid of functionalized aldimines **288** or **289** obtained by the condensation of 3-aminopyridine derivatives **285** with aldehydes containing a carbon–carbon double bond in *ortho* position **286** or **287** followed by prototropic tautomerization. The reaction transcurred in a selective manner allowing the generation of three stereogenic centers in a short fashion, and the *trans*-isomers **290** and **291** were obtained. The subsequent dehydrogenation of the fused tetrahydrochromeno[4,3-*b*][1,5]naphthyridines **290** and tetrahydrochromeno[4,3-*b*][1,5]naphthyridin-6-ones **291**, using DDQ as oxidant, leads to the formation of the corresponding tetracyclic chromeno[4,3-*b*][1,5]naphthyridine derivatives **292** and chromeno[4,3-*b*][1,5]naphthyridin-6-ones **293** in excellent yields (Scheme 83). The use of 2-aminopyridine derivatives, as amine component, afforded the corresponding 1,8-naphthyridine regioisomers [107]. The behavior as topoisomerase I inhibitors of the synthesized 1,5- and 1,8-naphthyridine derivatives was also studied.

**Scheme 84:**
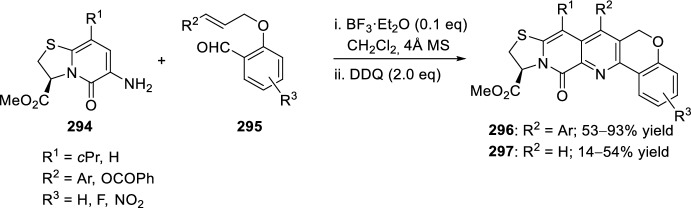
Synthesis of thiazolino-2-pyridone-based polyheterocycles capable of modulating and binding to α-synuclein and amyloid-β fibrils

A BF_3_·OEt_2_-catalyzed intramolecular Povarov reaction, followed by oxidation with DDQ, was used to synthesize chromenopyridine-fused thiazolino-2-pyridone peptidomimetics with the ability to bind α-synuclein and amyloid-β fibrils in vitro [108]. The reaction works with several *O*-alkylated salicylaldehydes **295** and amino functionalized thiazolino-2-pyridones **294**, to generate polyheterocycles **296** with diverse substitution in moderate to excellent yields (Scheme 84). On the contrary, attempts to synthesize C-7 unsubstituted molecules **296** (R^2^ = H) through intramolecular Povarov reaction, using *O*-allylsalicylaldehyde **295** (R^2^ = H) took place in very low yields, but the use of a vinyl ester moiety as electron-donating auxiliary **296** (R^2^ = OCOPh), allowed the obtainment of the C-7 unsubstituted compounds **297** in reasonable reaction times and moderate yields after removal of benzoate functionality during the oxidation process.

**Scheme 85:**
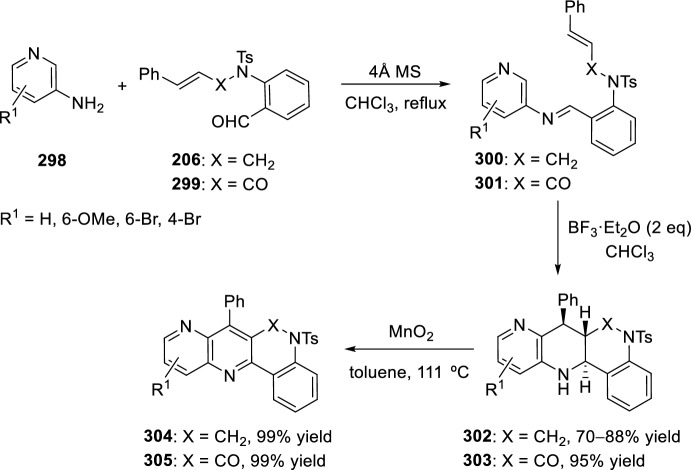
Synthesis of quinolino[4,3-*b*][1,5]naphthyridines and quinolino[4,3-*b*][1,5]naphthyridin-6(5*H*)-ones

Following the intramolecular strategy, hybrid substituted quinolino[4,3-*b*][1,5]naphthyridines **304** and quinolino[4,3-*b*][1,5]naphthyridin-6(5*H*)-ones **305** were synthesized [109]. The derivatives were achieved by an intramolecular Povarov [4 + 2]-cycloaddition reaction using BF_3_·OEt_2_ as Lewis acid (Scheme 85). First, the corresponding 5-tosyl functionalized aldehydes **206** (X = CH_2_) or **299** (X = CO), which tailored a double bond in their structure, condensed with 3-aminopyridines **298** to afford imines **300** or **301**, respectively. Subsequent regio- and stereospecific intramolecular cyclization in refluxing chloroform and in the presence of a Lewis acid such as BF_3_·OEt_2_ and prototropic tautomerization, gave the corresponding tetrahydro 1,5-naphthyridines **302** or 1,5-naphthyridin-6(5*H*)-ones **303** respectively, as *trans*-diastereoisomers. Their dehydrogenation reaction was performed using MnO_2_ in toluene-yielding compounds **304** or **305**. The corresponding deprotection of the tosyl group could be accomplished with magnesium under acidic conditions. The corresponding 1,8-naphthyridine regioisomers could also be prepared when 2-aminopyridine derivatives are used as the amine component in this intramolecular Povarov reaction [107].

**Scheme 86:**
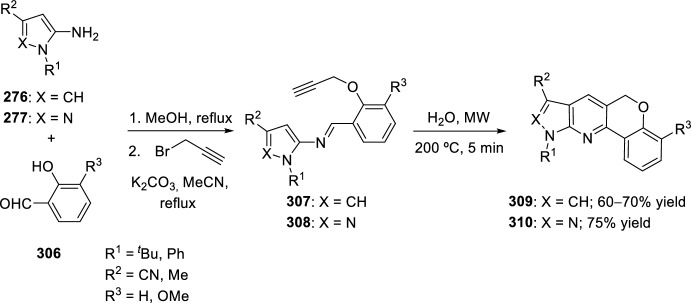
Intramolecular Povarov reaction using 2-aminopyrrole or 2-aminopyrazole and alkyne-tethered aldehydes

The previously reported methodology using 2-aminopyrrole **276** and 2-aminopyrazole **277** to construct chiral tetracyclic hexahydrochromenopyrrolo- and hexahydrochromenopyrazolo-pyridines (vide supra Scheme 81) was extended to alkyne bridged aldehydes derived from alkylation of aldehydes **306**. Thus, when propargyl bromide was used as the dienophile, the aromatic annulated compounds **309** or **310** were obtained in good yields via spontaneous aromatization of the corresponding cycloadducts (Scheme 86) [103].

**Scheme 87:**
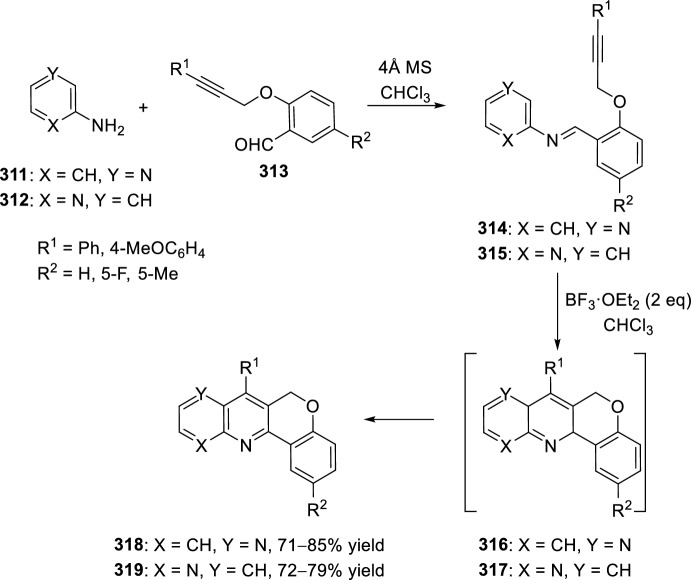
Synthesis of chromeno[4,3-*b*][1,5] and [1,8]naphthyridines using alkyne-tethered aldehydes

Similarly, aldimines **314** or **315**, derived from the condensation of substituted 2-propargyloxybenzaldehydes **313** and 3-aminopyridine **311** or 2-aminopyridine **312**, respectively, afforded the corresponding chromeno[1,5]naphthyridine derivatives **318** or chromeno[1,8]naphthyridine compounds **319** after BF_3_·OEt_2_-catalyzed intramolecular Povarov reaction (Scheme 87). It is noteworthy that, with this strategy, from a preparative point of view, the aromatic 1,5- and 1,8-naphthyridine core may be directly obtained [106, 107].

**Scheme 88:**
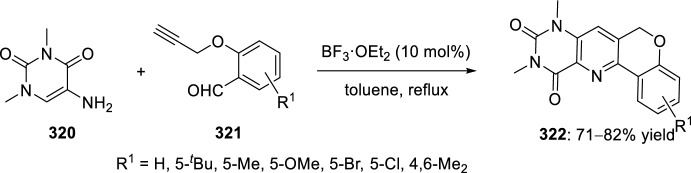
Intramolecular Povarov reaction involving 5-amino-1,3-dimethyl uracil for the preparation of chromenopyrido[3,2-*d*]pyrimidines

5-Amino-1,3-dimethyl uracil **320** has been used as the amino component in the intramolecular Povarov reaction. Thus, Majumdar et al. [110] reported in 2010 the Lewis acid catalytic intramolecular Povarov reaction between *O*-propargylated salicylaldehydes **321** and 5-amino-1,3-dimethyl uracil **320** (Scheme 88). Several Lewis acids (BF_3_·OEt_2_, Yb(OTf)_3_, CuBr, and CuI), Brønsted acids (TFA), and solvents (MeCN, THF, DMF, DMSO, EtOH, and toluene) were screened. All these variations of the catalyst and solvent showed that running the reaction in toluene using 10 mol% of BF_3_·OEt_2_ as the catalyst provides the best results for the synthesis of chromene-fused pyrido[3,2-*d*]pyrimidines **322** in good chemical yields (Scheme 88).

**Scheme 89:**
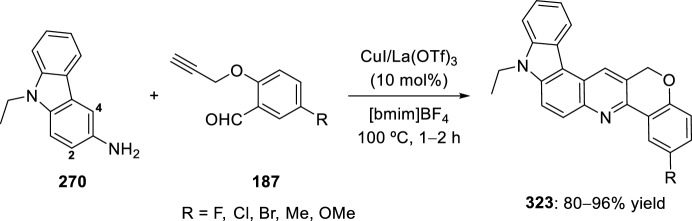
Intramolecular Povarov reactions in the synthesis of isoellipticine fused with dihydrochromene derivatives

Nagarajan et al. [111] have reported the synthesis of isomeric isoellipticine derivatives through a straightforward CuI/La(OTf)_3_-catalyzed tandem reaction in ionic liquid [bmim][BF_4_]. Thus, the reaction of bromo-, fluor-, chloro-, methyl-, or methoxy-substituted *O*-propargylated salicylaldehyde **187** with carbazole-derived amine **270** in the presence of CuI/La(OTf)_3_ and in ionic liquid afforded isoellipticine fused with dihydro chromene derivatives **323** in 80–96% chemical yield (Scheme 89). After careful analysis, authors identified the intramolecular Povarov occurred through C–4 of the carbazole ring.

**Scheme 90:**
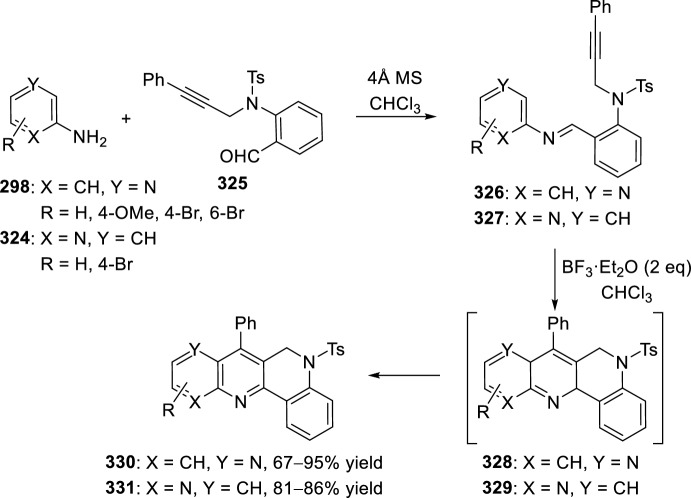
Synthesis of quinolino[1,5]naphthyridines and quinolino[1,8]naphthyridines

The synthesis of hybrid substituted quinolino[1,5]naphthyridines **330** and quinolino[1,8]naphthyridines **331** may also be obtained in good yields by BF_3_·OEt_2_-catalyzed intramolecular cycloaddition of aldimines **326** or **327**, derived from *N*-propargyl substituted aldehyde **325** and the corresponding aminopyridines **298** or **324** (Scheme 90) [107, 109]. The deprotection of the tosyl group at the nitrogen atom was carried out with Mg under acidic conditions.

See Table 5 for the most representative examples of Sect. 6.

**Table 5:** Some examples of the intramolecular Povarov reaction between heteroaromatic amines and aromatic aldehydes

Entry	Heteroaromatic amine	Aromatic aldehyde	Catalyst	Compound	dr *cis/trans*	Yield (%)	References
1			No catalyst		0:100	62	[105]
2			No catalyst		0:100	67	[105]
3			No catalyst		100:0	68	[105]
4			La(OTf)_3_		95:5 to 98:28	85–93	[104]
5			BF_3_·OEt_2_		–	71–85	[106]
6			BF_3_·OEt_2_		–	72–79	[107]
7			BF_3_·OEt_2_		–	71–82	[110]
8			CuI/La(OTf)_3_		–	80–96	[111]
9			BF_3_·OEt_2_		–	67–95	109]
10			BF_3_·OEt_2_		–	81–86	[107]

## Heteroaromatic Amines and Heteroaromatic Aldehydes

**Scheme 91:**
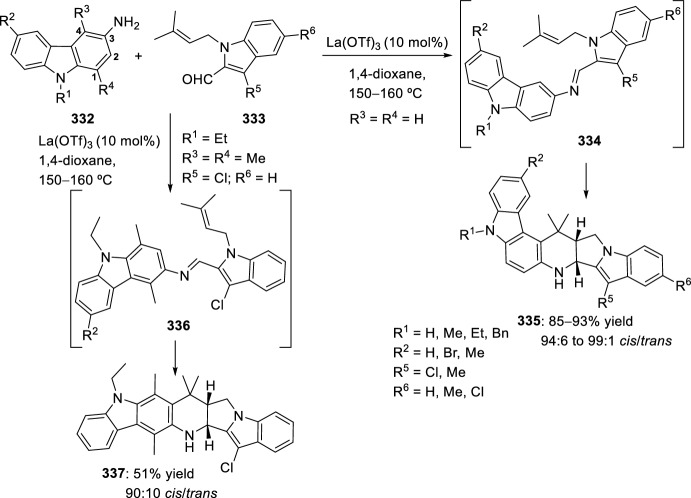
Synthesis of isomeric ellipticine derivatives by means of intramolecular Povarov reaction of heteroaromatic aldehydes and heteroaromatic amines

Only one report has disclosed in the intramolecular Povarov reaction of imine intermediates derived from heteroaromatic aldehydes and heteroaromatic amines. In this way, Nagarajan et al. [104] demonstrated the utility of imines, resulting from the condensation of *N*-prenylated indole-2-carbaldehydes **333** with aminocarbazoles **332**, in the intramolecular Povarov reaction. This protocol efficiently proceeds in the presence of Lewis or Brønsted acids, but diastereoselectivity is influenced by the nature of the catalyst. The best catalytic conditions were found for La(OTf)_3_ (10 mol%) in 1,4-dioxane at 150–160 °C, yielding isomeric ellipticine ring system derivatives **335** (R^3^ = R^4^ = H) in good yields and excellent diastereoselectivities (Scheme 91). The intramolecular Povarov reaction occurred through C–4 of the carbazole ring, and the six-membered piperidine and five-membered pyrrolidine rings were *cis*-fused. When amine **332** with substitution at C–1 and C–4 (R^3^ = R^4^ = Me) reacted with aldehyde **333**, the intramolecular cyclization occurred through the C–2 position of the carbazole ring, affording the corresponding product **337** in only 51% yield (Scheme 91).

## Intramolecular Povarov Reaction with Secondary Amines

**Scheme 92:**
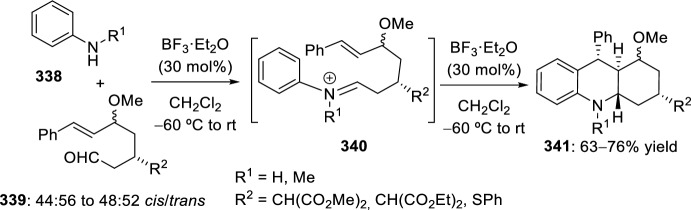
Intramolecular Povarov reaction of iminium ions for the synthesis of octahydroacridines

In 1996 Beifuss’s group [112] developed the first intramolecular cationic Povarov cyclization. The condensation of *N*-substituted anilines **338** and chiral ω-unsaturated aldehydes **339** in situ affords cationic iminium ions **340** (Scheme 92). Subsequent intramolecular [4 + 2]-cycloaddition of **340** leads to the highly *trans*-diastereoselective formation of octahydroacridines **341** with five stereogenic centers. The best yields were obtained when the transformation was performed with BF_3_·OEt_2_ (30 mol%) as the Lewis acid in CH_2_Cl_2_.

**Scheme 93:**
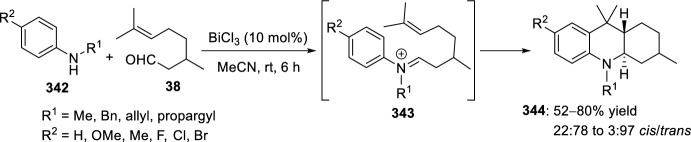
BiCl_3_-catalyzed intramolecular cationic Povarov reaction for the construction of octahydroacridines

A one-pot diastereoselective synthesis of new *N*-substituted octahydroacridines was successfully achieved by Kouznetsov et al. [113, 114] via BiCl_3_-catalyzed intramolecular cationic imino-Diels–Alder reaction. The intermediate iminium ions were prepared in situ through condensation of *N*-protected anilines **342** and (±)-citronellal **38** under mild reaction conditions (Scheme 93). It was observed that bulky *N*-substituent groups play a key role in the *cis*/*trans* ratio of the corresponding octahydroacridines **344**. For instance, when R^1^ = Me, a mixture of 50:50 *cis*/*trans*-octahydroacridine derivatives **344** were observed. However, increasing bulkiness (R^1^ = allyl, propargyl, and benzyl substituents) allows preferential formation of the *trans*-fused heterocycles in ratios ranging from 22:78 to 3:97 *cis*/*trans*-isomers. It was found that use of the *N*-benzyl group resulted in a highly diastereoselective process that gives easily separable *trans*-fused *N*-substituted octahydroacridines **344**. The developed protocol was extended to involve the use of citronella essential oil from *Cymbopogon* *nardus* as a renewable source of these biologically important heterocyclic molecules. The results obtained from *C.* *nardus* essential oil are comparable to those observed from pure (±)-citronellal **38**, without changes in the diastereoselectivity. Furthermore, this methodology can be extended to other *N*-substituted anilines with reactive groups as starting materials to prepare different *trans*-*N*-substituted hybrid octahydroacridines **344** as potential bioassay substrates.

**Scheme 94:**
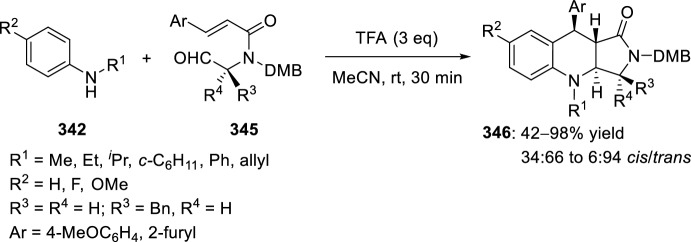
Intramolecular cationic Povarov reaction catalyzed by TFA in the preparation of pyrroloquinolinones

Spaller et al. [115] described the application of the intramolecular aza-Diels–Alder transformation to generate a diverse range of quinoline-fused structures with multiple stereogenic centers, many of which resemble lignan and arylnaphthalene-type natural products. In this work, they combined several secondary (*N*-alkylated) anilines **342** and aldehyde-alkene bridge **345**, with various Brønsted and Lewis acid catalysts (Scheme 94). For instances, several Brønsted acids such as TFA, trifluoromethanesulfonic acid (TFMSA), acetic acid, *p*-TsOH, or l-tartaric acid and Lewis acids such as Yb(OTf)_3_ or BiCl_3_ were used for this transformation. The results demonstrated that both Brønsted and Lewis acids have a slight effect on both selectivity and overall yields. The use of 3 equivalents of TFA in acetonitrile at room temperature afforded hexahydropyrrolo[3,4-*b*]quinolin-1-ones **346** in moderate to excellent yields, and diastereoselectivities ranging from 34:66 to 6:94 (Scheme 94).

**Scheme 95:**
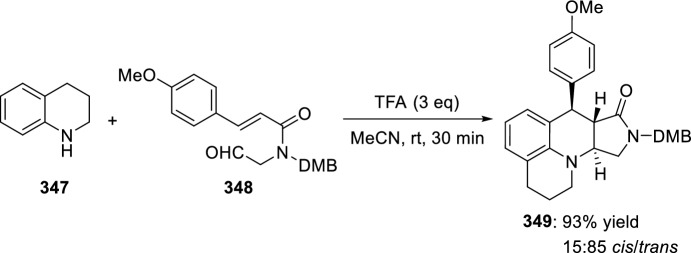
Synthesis of diazacyclopenta[*a*]phenalenone by intramolecular cationic Povarov approach

*N*-alkyl substitution in the *N*-aromatic amine component was rigidified by incorporating a saturated ring system. Thus, trifluoroacetic acid-catalyzed intramolecular Povarov reaction of the corresponding iminium ion intermediate, generated in the condensation between 1,2,3,4-tetrahydroquinoline **347** and aldehyde **348**, bearing an alkene-tethered partner, produced cycloadduct **349** in 93% yield and 15:85 in favor of *trans*-selectivity (Scheme 95) [115].

**Scheme 96:**
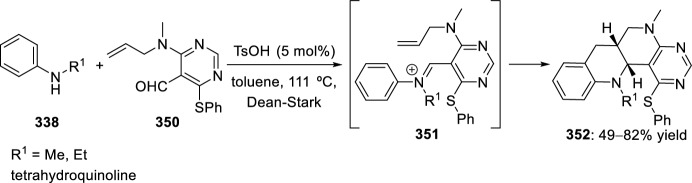
Intramolecular Povarov reaction in the preparation of benzopyrimido[4,5-*h*][1,6]naphthyridine libraries

Bai’s group [90] developed an efficient synthesis of tetracyclic pyrimidine-fused heterocycles through the intramolecular Povarov reaction of iminium salts **351** formed in situ from the reaction of secondary aryl amines **338** and allylaminopyrimidine-5-carbaldehyde **350**. *p*-Toluenesulfonic acid was selected as Brønsted acid catalyst, yielding solely *cis*-benzopyrimido[4,5-*h*][1,6]naphthyridines **352** in moderate to good yields (Scheme 96).

See Table 6 for the most representative examples of Sect. 8.

**Table 6:** Some examples of the intramolecular Povarov reaction using secondary amines

Entry	Amine	Aldehyde	Catalyst	Compound	dr *cis/trans*	Yield (%)	References
1			BF_3_·OEt_2_		0:100	63–76	[112]
2			BiCl_3_		22:78 to 3:97	52–80	[113, 114]
3			TFA		34:66 to 6:94	42–98	[115]
4			TFA		15:85	93	[115]
5			TsOH		100:0	49–82	[90]

## Oxidative Intramolecular Povarov Reaction

In 2011, Mancheño’s group described the oxidative Povarov reaction between glycine derivatives with olefins [116]. In this method, a crucial oxidant was required for both the in situ generation of the iminium intermediate (by a C_sp3_–H bond oxidation of *N*-aryl amine) and the final dehydrogenation of the tetrahydroquinoline to form the corresponding heteroaromatic compound. The strategy represents a milestone in organic synthesis and specifically in the Povarov reaction as no prefunctionalization is required in the reaction partners and CH bonds are ubiquitous in organic molecules. Since then, the oxidative Povarov reaction to form quinoline derivatives, also in its intramolecular version, has been extensively studied as the process involves the use of simpler starting materials and less waste generation.

**Scheme 97:**
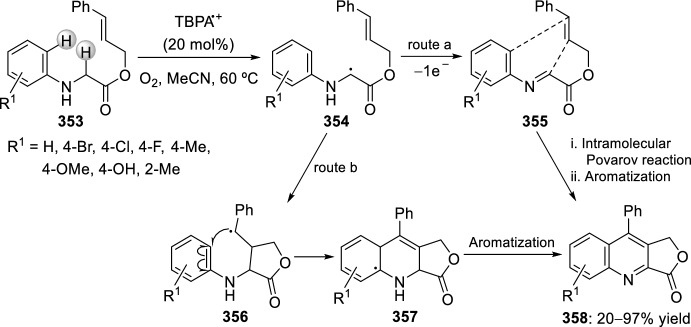
Catalytic radical cation salt induced C_sp3_–H oxidation for the construction of quinoline-fused lactones

C_sp3_–H bond oxidation of *N*-aryl glycine esters and amides can be carried out under catalytic radical cation salt-induced conditions. The peroxyl radical cation, which is generated in the coupling between tris(4-bromophenyl)ammoniumyl hexachloroantimonate (TBPA^+^·) and oxygen, might be involved to initiate the catalytic oxidation. In this way, Jia’s group [117, 118] reported a direct construction of quinoline-fused lactones accomplished by C_sp3_–H bond oxidation under catalytic radical salt-induced conditions of starting *N*-aryl glycine esters. Radical cation salt TBPA^+^, stable in the solid state but decomposed after 3 h in MeCN in the presence of oxygen, promotes the cyclization of glycine derivatives **353** to yield Povarov adducts. Both electron-withdrawing and electron-donating substituted *N*-aryl glycine cinnamyl esters **353** were converted to quinoline-fused lactones **358** in moderate to excellent yields (Scheme 97). The lack of substituents or the presence of a substituent at the *ortho* position of the aniline dramatically diminished the reaction yield; for instance, when R^1^ = H compound **358** was obtained in 49% and only 20% yield of **358** was observed when R^1^ = 2-Me. The key step in this transformation comprises the formation of a glycine imine (or iminium ion) that can efficiently add to the styrene moiety to afford Povarov adducts. In this way, authors suggest a plausible mechanism where the sp^3^ C–H bond adjacent to the aniline group is oxidized by TBPA^+^· in the presence of oxygen, giving radical intermediate **354**, which can be further oxidized to the corresponding glycine imine **355** (Scheme 97, route a). The formation of quinoline-fused lactones **358** may arise from a radical cation salt-induced Povarov reaction. Nevertheless, radical intermediate **354** may also add to the double bond of styrene, and subsequent radical addition to the phenyl group would yield **357**. Further oxidation and aromatization would afford **358** (Scheme 97, route b) [118].

**Scheme 98:**
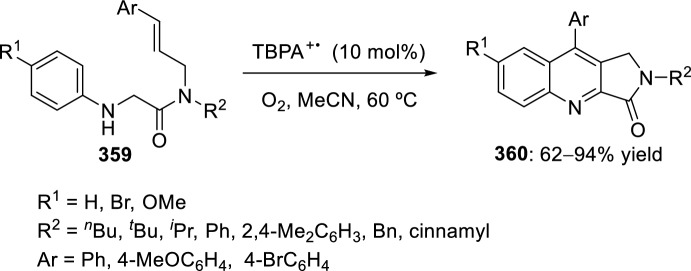
Catalytic radical cation salt induced C_sp3_–H oxidation for the construction of quinoline-fused lactams

The generality of this protocol was determined by the use of glycine amides toward the construction of quinoline-fused lactams **360** under the same catalytic radical salt-induced conditions [118]. All of the tested *N*-aryl glycine cinnamyl amides **359** displayed good reactivity, yielding the corresponding quinoline-fused lactams **360** in good to excellent yields (Scheme 98). Bulky amide *N*-protecting groups gave better results, probably due to the closeness of the cinnamyl group to the reactive radical in those derivatives, which favors the intramolecular annulation. In addition, the effect of the substituents on the cinnamyl group was studied, showing that electron-donating groups increase the annulation yields.

**Scheme 99:**
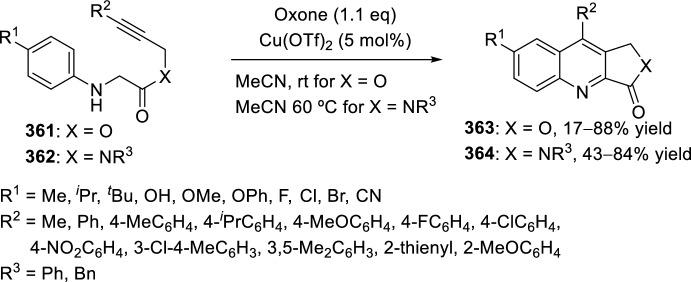
Oxone promoted intramolecular dehydrogenation followed by Povarov cyclization for the construction of quinoline-fused lactones and lactams

More recently, Muthukrishnan’s group [119] reported an intramolecular dehydrogenation promoted by oxone followed by imino-Diels–Alder reaction (Povarov cyclization) of alkyne-tethered *N*-aryl glycine esters and amides for the preparation of quinoline-fused lactones and lactams. Hence, the dehydrogenative Povarov reaction of *N*-aryl glycine ester **361** (X = O) was proved by using 5 mol% BF_3_·OEt_2_ as a Lewis acid in the presence of 2-iodoxybenzoic acid (IBX) as an oxidant at room temperature. Under these conditions, quinoline fused lactones **363** were obtained in 58% yield (Scheme 99). Other Lewis acids, such as Cu(OTf)_2_ or Sn(OTf)_3_, and different peroxide-based oxidants such as PhI(OAc)_2_, PhI(IOCOCF_3_)_2_, Na_2_S_2_O_8_, benzoyl peroxide (BPO) or Oxone (2KHSO_5_-KHSO_4_-K_2_SO_4_) were screened. The combination of Cu(OTf)_2_ as Lewis acid and Oxone as oxidant works well, affording cycloadducts **363** in good yields, except for methyl-substituted alkyne **361** (R^2^ = Me), which was obtained in 17% yield (Scheme 99). Remarkably, Oxone would be a favorable oxidant as it is easy to handle, cheap, and nontoxic. The scope and generality of this approach indicated that different electron-donating and electron-withdrawing groups on the aniline ring as well as the aryl alkyne moiety were well tolerated (Scheme 99). This method was further extended to the preparation of quinoline-fused lactams **364** (X = NR^3^) (Scheme 99). The intramolecular dehydrogenative Povarov cyclization of *N*-aryl glycine amide **362** (X = NR^3^) using the optimized reaction conditions, yielded the cycloadducts **364** in moderate to good yield, although higher temperatures were required for reaction completion [119]. This protocol is very general since electron-donating and electron-withdrawing groups on the aniline ring as well as the aryl alkyne moiety of NR^3^ protected glycine amide furnished the corresponding products **364** (Scheme 99). The method was further used for the preparation of biologically important quinoline core of uncialamycin and luotonin A analogs.

## Asymmetric Intramolecular Povarov Reaction

**Scheme 100:**
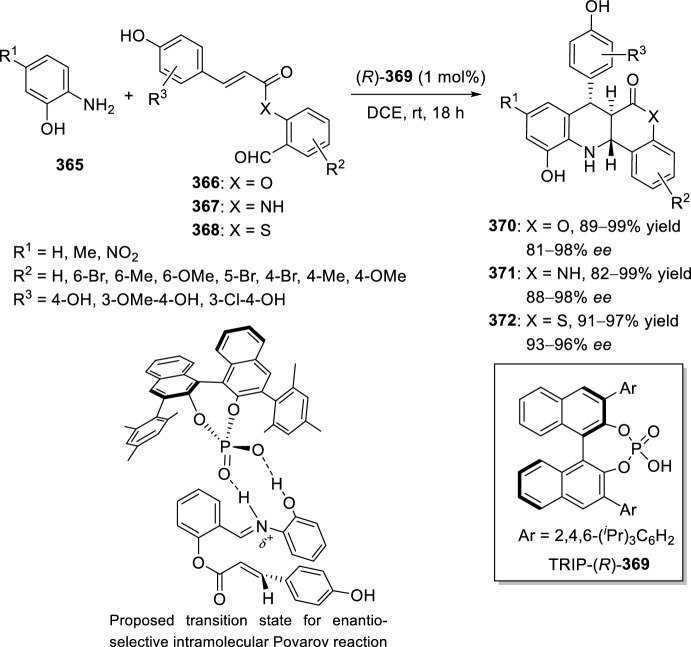
TRIP-catalyzed enantioselective organocatalytic intramolecular Povarov reaction

Masson’s group first described the asymmetric version of the intramolecular Povarov reaction in 2017 [120]. They developed an efficient asymmetric organocatalytic intramolecular Povarov reaction for the preparation of optically active chromeno-fused quinoline derivatives as well as dibenzo-fused naphthyridine derivatives. A chiral phosphoric acid, (*R*)-3,3′-bis(2,4,6-triisopropylphenyl)-1,1′-binaphthyl-2,2′-diyl hydrogen phosphate (**369**, TRIP) catalyzes the enantioselective intramolecular Povarov-type reaction of alkene-tethered aldehydes **366**–**368** and primary 2-hydroxy anilines **365**. The corresponding tetrahydrochromeno[4,3-*b*]quinolin-6-ones **370** (X = O) as well as tetrahydrodibenzo[1,6]naphthyridin-6-ones **371** (X = NH) were obtained in excellent yields, with high diastereo- and enantioselectivities ranging from 81% to 98% *ee*s (Scheme 100). The intramolecular cycloaddition proceeds at room temperature with complete diastereoselectivity in favor of the *trans*,*trans*-tetrahydrochromeno[4,3-*b*]quinolin-6-one derivative **370** when the dienophile possesses a benzene ring with a hydroxy group at *para*-position, while *ortho*- and *meta*-analogs were completely unreactive in this cycloaddition. It appears to be reasonable that hydrogen bonding of the phosphoric acid **369** to *p*-phenol seems to be critical for effective cycloaddition, but not for enantioselectivity, since 35% *ee* has been observed for **370** when 4-methoxy aniline was used. The use of 2-aminophenol **365** (R^1^ = H) as starting material might form an additional hydrogen bond with the chiral phosphoric acid **369** to increase facial discrimination of the *N*-2-hydroxy aldimine in the Povarov cycloaddition. This is observed by a dramatic increase in enantioselectivity. Furthermore, the catalyst loading could be lowered to 1 mol%, and the obtained azacycles were formed in high yield and high purity, after precipitation in the reaction vessel and isolation by filtration without purification by column chromatography. As an extension of a previous paper, Masson et al. [121] reported the enantioselective intramolecular Povarov synthesis of tetrahydrothiochromeno[4,3-*b*]quinolin-6-ones **372** (X = S, Scheme 100). These new fused nitrogen-containing tetraheterocycles were attained in excellent diastereo- and enantioselectivity (93–96% *ee*), although the reaction conversion is not completed. However, a slight increase in the catalyst loading to 2 mol% gave the corresponding cycloadducts in excellent yields (91–97%). If the intermediate imine coordinates in a bidentate manner to the chiral phosphoric acid (*R*)-**369** through a nine-membered cyclic transition state, the cyclization pathway may proceed by a stepwise mechanism (Scheme 100).

**Scheme 101:**
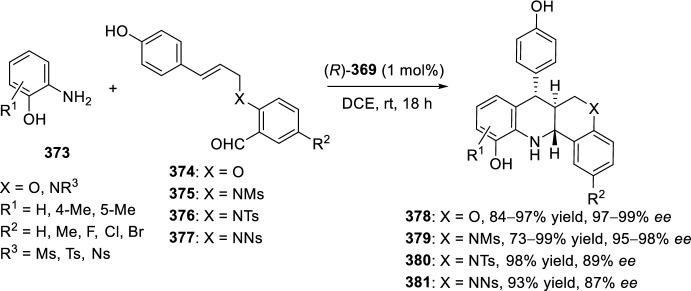
Asymmetric intramolecular Povarov reaction for the preparation of enantiomerically enriched tetrahydrochromeno[4,3-*b*]quinolines and hexahydrodibenzo[*b*,*h*][1,6]naphthyridines

To further demonstrate the efficiency and scope of the present method, it was next applied to linear precursors with ether (**374**, X = O) or amine groups (**375**–**377**, X = NR^2^) as the linker between the aromatic aldehyde ring and the styrene group [121]. Precursors with an ether group as a linker were smoothly converted into the corresponding tetrahydro-6*H*-chromeno[4,3-*b*]quinolines **378** in excellent yields, enantioselectivities up to 99% *ee*, and *trans*,*trans*-diastereoselectivities (Scheme 101). In the same way, linear precursors with amine linker provided access to hexahydrodibenzo[*b*,*h*][1,6]naphthyridines **379**–**381**, which were obtained with excellent yields, diastereo- and enantioselectivities ranging from 87% to 98% *ee* (Scheme 101). Diversely substituted 2-aminophenols were suitable partners to afford the corresponding cycloadducts in high yields and enantioselectivities. Modification of the protecting group on nitrogen afforded the desired products with similar yields although with slightly lower enantioselectivity.

**Scheme 102:**
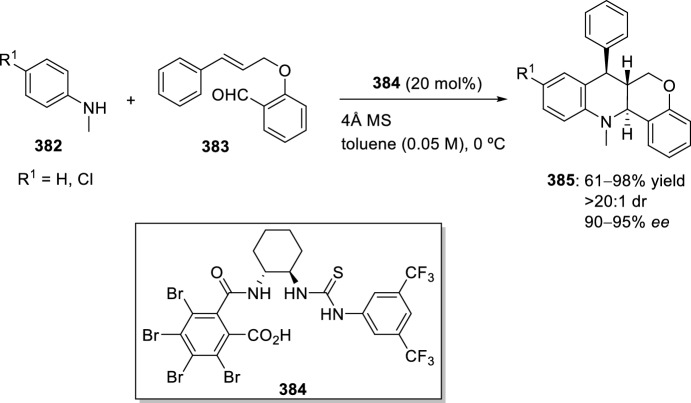
Enantioselective intramolecular Povarov reaction with secondary anilines

Catalytic enantioselective reactions with secondary amine substrates that involve intermediate iminium ions remain far less studied than the corresponding transformations with primary amines or preformed imines. Seidel et al. [122], for the purpose of developing the such transformation, synthesized polycyclic amines containing three contiguous stereogenic centers with excellent stereocontrol in a single step from *N*-methyl aryl amines **382** and aldehyde **383** possessing a pendant dienophile. Thus, *N*-methyl aryl amines **382** and *O*-allylsalicylaldehyde derivative **383** reacted using chiral Brønsted acid catalyst **384**, afforded tetrahydrochromeno[4,3-*b*]pyrrolo[3,2,1-*ij*]quinolines **385** in moderate to excellent yields and high levels of diastereo- and enantioselectivity (Scheme 102). Catalyst **384**, which involves a carboxylic acid group and a thiourea moiety as a covalently connected anion-recognition site, was previously applied to a catalytic enantioselective three-component Povarov reaction [123].

**Scheme 103:**
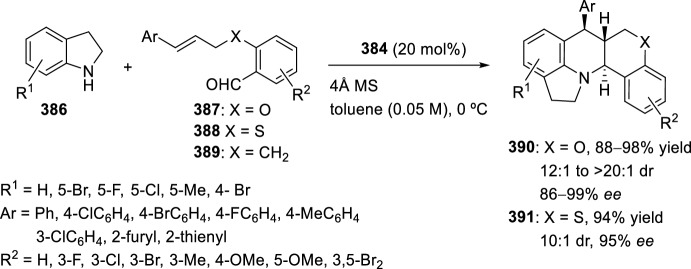
Enantioselective intramolecular Povarov reaction with indoline derivatives

Likewise, indoline derivatives **386** and *O*-allylsalicylaldehyde derivatives **387** with different substituents on the aldehyde phenyl ring, reacted using chiral Brønsted acid catalyst **384**, affording tetrahydrochromeno[4,3-*b*]pyrrolo[3,2,1-*ij*]quinolines **390** with excellent yields and high levels of diastereo- and enantioselectivity (Scheme 103) [123]. Similarly, the thiosalicylaldehyde-derivative **388** (X = S) was fruitfully converted into the corresponding tetrahydropyrrolo[3,2,1-*ij*]thiochromeno[4,3-*b*]quinoline **391** with a slight decrease of diastereomeric ratio; however, excellent yield and enantioselectivity was preserved (Scheme 103). The starting material in which the oxygen linker is replaced with a methylene bridge **389** (X = CH_2_) was similarly reactive but provides products in racemic form (dr = 2:1). Regarding to the amine component, tetrahydroquinoline reacted to afford the corresponding cycloadduct in 83% chemical yield after 2 days, but with reduced diastereoselectivity (dr = 7:1) and enantioselectivity (81% *ee*).

**Scheme 104:**
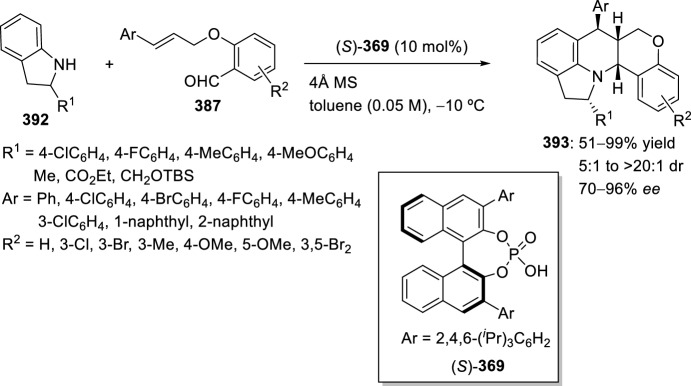
Enantioselective intramolecular Povarov reaction with two-substituted indoline derivatives

As an extension of Seidel’s methodology, the same group introduced a new approach for the rapid synthesis of polycyclic amines through aza-Diels–Alder (Povarov) reaction, applying the kinetic resolution of two-substituted indolines to enhance the stereochemical complexity of the products [124]. Under control of a chiral Brønsted acid catalyst, racemic 2-phenylindoline **392** (R^1^ = Ph) undergoes intramolecular Povarov reaction with achiral aromatic aldehydes **387** bearing a pendant dienophile. One enantiomer of the indoline reacts preferentially, resulting in the highly enantio- and diastereoselective formation of polycyclic heterocycles, tetrahydrochromeno[4,3-*b*]pyrrolo[3,2,1-*ij*]quinolines **393** with four stereogenic centers (Scheme 104). This kinetic resolution approach exploits the differential formation/reactivity of diastereomeric ion pairs. The use of (*S*)-TRIP **369** provided good results, which were increased lowering the temperature to –10 ºC, improving de enantioselectivity process. The scope of this transformation was evaluated with a range of *O*-allylsalicylaldehyde derivatives **387**. A range of substituents on the aldehyde phenyl ring and the styrene component were readily tolerated in reactions with 2-phenylindoline **392** (R^1^ = Ph), producing polycyclic heterocycles in excellent yields (82–99%), diastereo- (up to 20:1) and enantioselectivities (80–96% *ee*). Variation of the indoline cycloaddition partner was evaluated next. A number of two-substituted indolines **392** with diverse substituents (R^1^ = Ar, Alk) performed well. Even ethyl ester substituent or *tert*-butyldimethylsilyl (TBS)-protected alcohol were accommodated (Scheme 104).

**Scheme 105:**
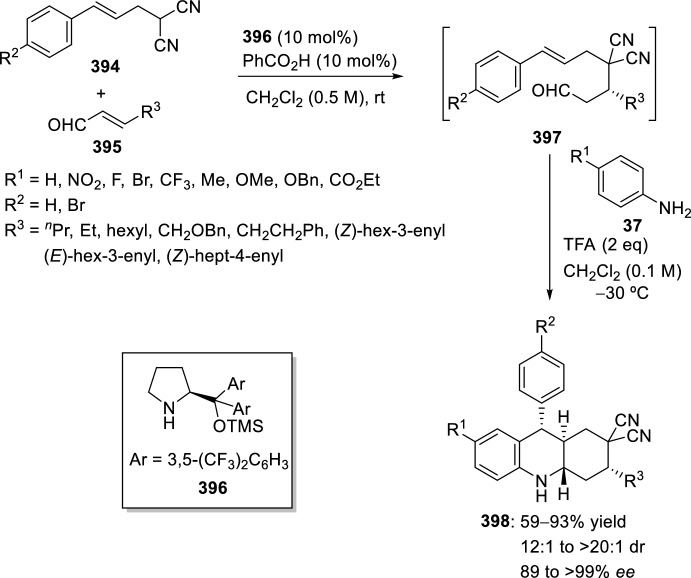
Enantioselective organocatalytic one-pot domino Michael/intramolecular Povarov reaction with malononitriles

Jørgensen’s group [125] developed an efficient asymmetric organocatalytic one-pot domino Michael addition/intramolecular Povarov reaction for the synthesis of optically active octahydroacridines having four stereocenters. Thus, malononitriles **394** react with a series of aliphatic α,β-unsaturated aldehydes **395** and *p*-substituted anilines **37**, in the presence of diarylprolinol **396** and benzoic acid as additive, to produce octahydroacridines **398** with high yields and excellent enantio- and diastereomeric control (Scheme 105). The conjugate addition of malononitriles **394** to α,β-unsaturated aldehydes **395** using aminocatalysis leads to the formation of proper intermediates **397**, which could be trapped in an amine condensation/intramolecular Povarov cascade to afford products **398**. This asymmetric organocatalytic one-pot domino protocol displays great tolerance toward different aliphatic α,β-unsaturated aldehydes, malononitriles, and *p*-substituted anilines.

**Scheme 106:**
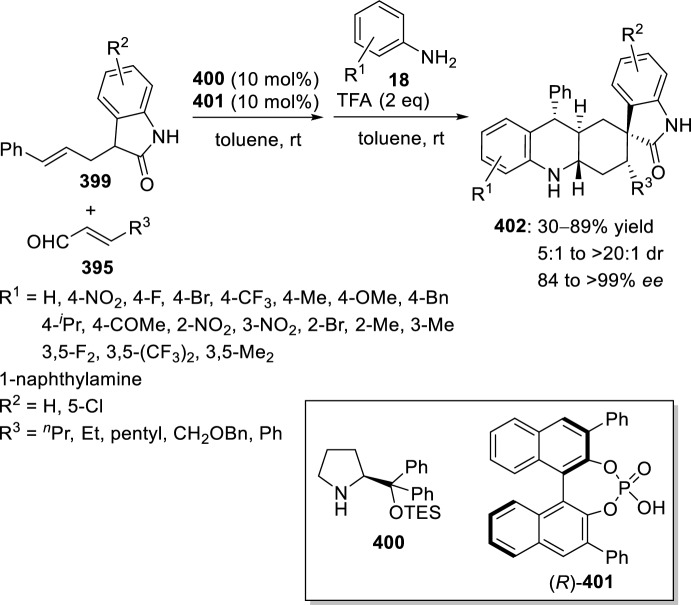
Enantioselective organocatalytic one-pot domino Michael/intramolecular Povarov reaction with indolinones

A similar strategy, settled by Wang’s group [126], entails an effective organocatalytic one-pot domino Michael/intramolecular Povarov reaction using substituted indolinones **399**, α,β-unsaturated aldehydes **395** and aromatic amines **18** (Scheme 106). Different commercially available chiral secondary amines were scrutinized, due to their recognized abilities to activate α,β-unsaturated aldehydes toward asymmetric transformation. The diarylprolinol **400** with bulky ether groups such as *O*-triethylsilyl (*O*-TES) led to enantiomerically enriched spirooctahydroacridine-3,3′-oxindoles **402** with the generation of five sterogenic centers at the same time with higher diasteroselectivities and enantioselectivities. Next, authors examined the addition of additives, reveling that the combination of diarylprolinol **400** with chiral Brønsted acid **401** gave products **402** in higher yield and excellent stereoselectivities. Under optimal conditions, the scope to probe the generality of this procedure was examined, exhibiting an excellent acceptance toward a variety of different substrates furnishing enantiomerically enriched spirooctahydroacridine-3,3′-oxindole **402** in good yields, diastereoselectivities up to 20:1, and enantioselectivities ranging from 84% to > 99% (Scheme 106).

## Conclusions

The Povarov reaction allowed the preparation of heterocyclic compounds, tetrahydroquinoline skeleton, in a chemo-, regio- and stereoselective way by [4 + 2] cycloaddition between aromatic aldimines and dienophiles, in the presence of Lewis or Brønsted acids and under mild reaction conditions. A particular type of Povarov reaction is its intramolecular version when both, the aromatic imine (diene) and the dienophile system, are present in the same initial chemical structure, so that in the presence of Lewis or Brønsted acids this strategy allows the synthesis of a wide variety of fused heterocyclic compounds. These present important applications in medicinal, biological, and materials chemistry.

The intramolecular Povarov reaction provides a simple way to modulate the structural variety of the heterocyclic compounds to be prepared. Thus, the review began with a description of the heterocycles formed from aromatic amines with aliphatic, aromatic, and heteroaromatic aldehydes, followed by an analysis of the heterocycles obtained from heteroaromatic amines with different aldehydes. Likewise, special sections have been devoted to the intramolecular Povarov reaction with secondary amines and to the oxidative Povarov reaction demonstrating the applicability of this tool. Finally, the enantioselective preparation of fused heterocycles has been also addressed. As an improvement of the methods described so far, further exploration remains to be done in this field to discover new synthetic strategies for the preparation of new molecules and the development of new efficient asymmetric catalytic protocols to obtain compounds in an enantioselective way.

## Data Availability

The data reported in this review article is available in the internet, as in the references stated below. The authors also confirm that the data supporting the findings of this study are available within the article.

## Abbreviations

AMEBA: Acid-sensitive methoxy benzaldehyde polystyrene
BA: Brønsted acid
bmim: 1-Butyl-3-methylimidazolium
BPO: Benzoyl peroxide
BPTA: 2,5-Bis-propargyloxy terephthalaldehyde
CAN: Ammonium cerium (IV) nitrate
COF: Covalent organic framework
*o*-DCB: *o*-Dichlorobenzene
DCE: 1,2-Dichloroethane
DCM: Dichloromethane
DDQ: 2,3-Dichloro-5,6-dicyano-*p*-benzoquinone
DMB: 2,4-Dimethoxybenzyl
DME: Dimethoxyethane
DMF: Dimethylformamide
DMSO: Dimethylsulfoxide
eq: Equivalents
FDA: Food and Drug Administration
hmim: 1-Hexyl-3-methylimidazolium
IBX: 2-Iodoxybenzoic acid
IL: Ionic liquids
LA: Lewis acid
LPDE: Lithium perchlorate in diethyl ether
MCF-7: Breast cancer cell line
MCR: Multicomponent reaction
MDA-MB-231: Breast cancer cell line
MIC: Minimum inhibitory concentration
Ms: Mesyl
MS: Molecular sieves
MW: Microwave
NiP: Ni-porphyrin
NMR: Nuclear magnetic resonance
Ns: Nosyl
octmim: 1-Octyl-3-methylimidazolium
Piv: Pivaloyl
QUIN: Quinoline
rt: Room temperature
TAPB: 1,3,5-Tris(4-aminophenyl)benzene
TAPT: 1,3,5-Tris(4-aminophenyl)triazine
TBAF: Tetra-*n*-butylammonium fluoride
TBPA: Tris(4-bromophenyl)ammoniumyl hexachloroantimonate
TBS: *tert*-Butyldimethylsilyl
*O*-TES: *O*-Triethylsilyl
TFA: Trifluoroacetic acid
TFE: Trifluoroethanol
TFMSA: Trifluoromethanesulfonic acid
THF: Tetrahydrofurane
THQ: Tetrahydroquinoline
*O*-TMS: *O*-Trimethylsilyl
TRIP: 3,3′-Bis(2,4,6-triisopropylphenyl)-1,1′-binaphthyl-2,2′-diyl hydrogen phosphate
TPP: Triphenylphosphonium perchlorate
Ts: Tosyl
TS: Transition state
